# Changes of Mind in an Attractor Network of Decision-Making

**DOI:** 10.1371/journal.pcbi.1002086

**Published:** 2011-06-23

**Authors:** Larissa Albantakis, Gustavo Deco

**Affiliations:** 1 Department of Information and Communication Technologies, Computational Neuroscience, Universitat Pompeu Fabra, Barcelona, Spain; 2 Institució Catalana de la Recerca i Estudis Avançats (ICREA), Universitat Pompeu Fabra, Barcelona, Spain; University of Oxford, United Kingdom

## Abstract

Attractor networks successfully account for psychophysical and neurophysiological data in various decision-making tasks. Especially their ability to model persistent activity, a property of many neurons involved in decision-making, distinguishes them from other approaches. Stable decision attractors are, however, counterintuitive to changes of mind. Here we demonstrate that a biophysically-realistic attractor network with spiking neurons, in its itinerant transients towards the choice attractors, can replicate changes of mind observed recently during a two-alternative random-dot motion (RDM) task. Based on the assumption that the brain continues to evaluate available evidence after the initiation of a decision, the network predicts neural activity during changes of mind and accurately simulates reaction times, performance and percentage of changes dependent on difficulty. Moreover, the model suggests a low decision threshold and high incoming activity that drives the brain region involved in the decision-making process into a dynamical regime close to a bifurcation, which up to now lacked evidence for physiological relevance. Thereby, we further affirmed the general conformance of attractor networks with higher level neural processes and offer experimental predictions to distinguish nonlinear attractor from linear diffusion models.

## Author Summary

A recent psychophysical experiment showed that participants do adjust their decisions (change their mind) based on further evidence, which was processed only after the first decision was made. The established notion of (perceptual) decision-making as a decision variable evolving in time until a termination criterion is reached does not incorporate these changes of mind. In the biophysically-realistic attractor model, the mean firing rates of neural populations encoding the decision alternatives act as the decision variable. In line with neurophysiological evidence from decision-related neurons in the lateral intraparietal cortex, a decision is made if a fixed firing rate threshold is crossed. We propose here that a change of mind is induced if this decision threshold is crossed a second time, namely by the neural population encoding the initially losing alternative, which thus overtakes the population that first crossed the decision threshold. Interestingly, we found this more likely to happen the further the system is pushed towards a regime where decision-making is no longer unambiguous, but both neural populations can fire at elevated rates. This, besides, corresponds to higher incoming activity and thus faster and less accurate decisions and suggests that the brain operates over the whole range of inputs enabling decision-making.

## Introduction

In our lives, we constantly are required to make decisions. Some of these decisions are irretrievable, while others are not binding and can be adjusted if we change our mind. The brain processes leading to decisions, have occupied neuroscientists during the last decades (reviewed in: [1], [2]). Perceptual decision-making paradigms, like the random-dot motion (RDM) task [3]–[5], were designed to study decision-making behavior and brain activity of decision-associated brain areas, like the dorsolateral prefrontal cortex and lateral intraparietal (LIP) cortex, in the simplest context. Traditionally, the decision process is regarded as a decision variable evolving in time, until a termination criterion is reached. Firing rates of LIP neurons gradually increase during motion-viewing in the RDM task and correlate with subjects' choices and reaction times [3], [6], making LIP activity a possible candidate for a neural decision variable. Recently, more complex aspects of decision-making received increasing attention, involving multiple choices [6], [7] or confidence [8] and also: What happens in our brains if we change our mind?

**Figure 1:**
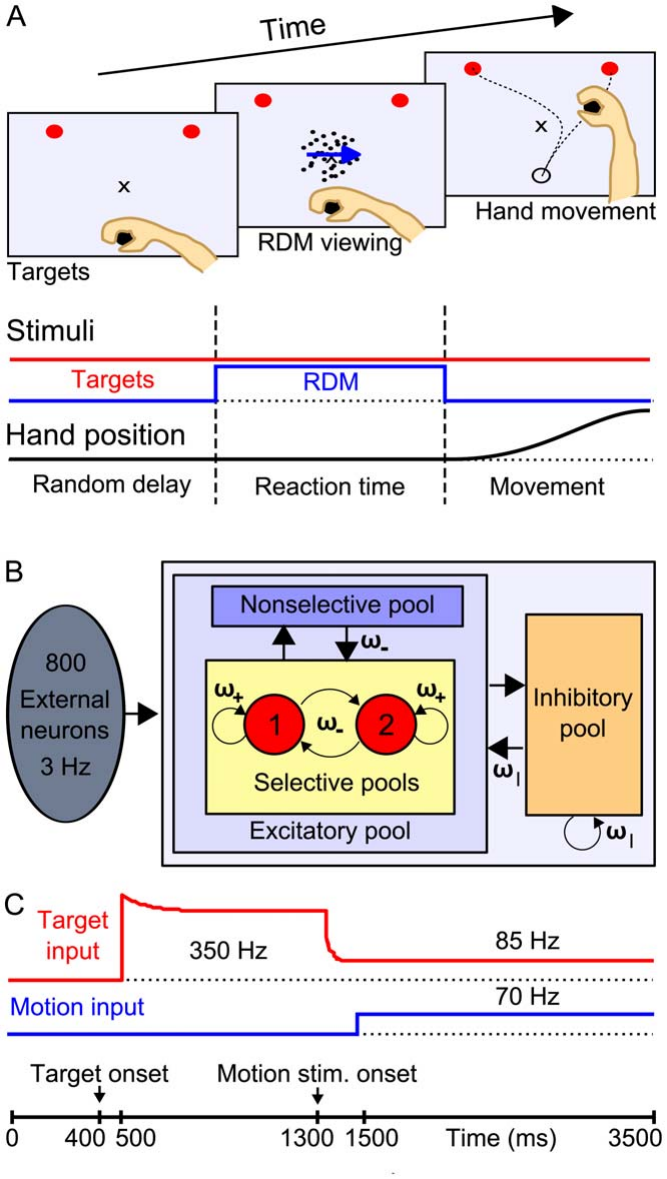
Experimental design, network architecture and stimulation protocol. (A) RDM paradigm with manual indication of choice as in Resulaj et al. [9]. See text for task details. In the majority of trials the subjects moved the handle directly to one of the targets. Some trajectories, however, revealed a change of mind during the movement: they started towards one direction but terminated at the opposite target. (B) Diagram of the binary attractor model for decision-making [14]. The network consists of a population of excitatory pyramidal neurons, structured into 2 selective pools (red, each contains 20% of the excitatory neurons) and a nonselective population, that inhibit each other through shared feedback from an inhibitory pool of interneurons (orange). Unlabeled arrows denote a connectivity of 1 (baseline). Recurrent connectivity within a selective pool is high, ω_+_ = 1.51, whereas the connection weight between the selective pools is below average ω_−_ = 0.8725. Inhibitory connections have a weight ω_I_ = 1.125. The network consists of 1,000 Neurons. (C) Time course of target and motion input to the selective populations in order to model the experimental design of the RDM task. The target input starts with a latency of 100 ms, the motion signal 200 ms after the respective stimulus onset (see methods).

To elucidate this question, Resulaj et al. [9] developed a psychophysical RDM task, where humans had to indicate their choice by moving a handle towards a left or right target (Fig. 1A). Because this hand movement is continuous, contrary to ballistic saccades or pressing a button [10], changes of mind could be directly observed by recording the handle traces. Changing improved the overall accuracy, but depended on task difficulty: most correcting changes were observed at intermediate levels, while erroneous changes increased monotonically with difficulty.

These findings pose a challenge for a class of models that implement decision-making by diffusion in a nonlinear landscape of stable fixed points, which act as decision-attractors. Once a decision-attractor is reached, this state will persist except for high levels of noise or perturbations and is thus rather counterintuitive to a change of mind. On the other hand, due to the stable attractors, those models account for persistent activity frequently observed in decision-related neurons. Moreover, biophysically-realistic attractor models, as introduced by Brunel and Wang [11], successfully simulate animal behavior and neural activity of LIP neurons during various versions of the RDM task [2], [12]–[14].

Here we show that changes of mind (after a first decision) are entirely consistent with attractor dynamics. In particular, they arise naturally during the itinerant transients following sensory perturbation, if the system lies close to a bifurcation (or phase boundary) that separates a neuronal state of categorical decision-making from a multi-stable region. There, the decision process is impeded by a second attractor, where both populations encoding the possible alternatives fire at high rates. This facilitates changes of mind. Moreover, by replicating the psychophysical data of Resulaj et al. [9] with a biophysically-realistic attractor network with spiking neurons, we gained neurophysiological predictions on neural firing rates during the change process. In all, our results offer testable predictions on the attractor concept and general principles of decision-making like the speed-accuracy trade-off and a fixed decision threshold.

## Results

With the objective to gain understanding of the actual brain processes during changes of mind, in the following we apply a biologically-inspired cortical model, first introduced by X.J. Wang [14], to the psychophysical findings of Resulaj et al. [9]. The experimental task sequence is illustrated in Fig. 1A. While the human participants were holding a handle at the starting position, a patch of randomly moving dots appeared after a random delay (0.7–1.0 s). Depending on the trial difficulty, a certain percentage of these dots were moving coherently to the left or right. The subjects had to decide within 2 s on the net direction of dot-motion and to report their choice by moving the handle towards a target (Fig. 1A, red dots) in the corresponding direction, within a time limit of 700 ms after they initiated the hand movement. Importantly, the moving-dot display was switched off when the handle left the starting position, which also determined the reaction time. Although the motion stimulus was no longer visible, on the way towards the target participants occasionally changed their mind and turned to the opposite target [9].

In the present attractor model, the two decision alternatives are implemented by two subpopulations (pools) of excitatory neurons, each selective for one of the two target directions (Fig. 1B, red). The decision process corresponds to the transition from a symmetric state, where both selective populations fire with about equal rates, to a decision state where they compete with each other in a winner-take-all manner, resulting in one pool firing at higher (winner), the other at lower rates (loser). The stability of the different attractor states (or fixed points) depends on the amount of input applied to the selective pools and the recurrent connectivity of the network populations. Consistent with a Hebbian rule, neurons within one selective pool have strong recurrent connections ω_+_, as their activity was supposedly correlated in the past, while the connections between selective pools are weaker than average ω_−_<1. A nonselective excitatory population represents activity of surrounding LIP neurons that are not selective to either direction. Competition arises in the network due to global feedback inhibition by a population of inhibitory neurons, connected to all neurons with weight ω_I_. To accurately simulate LIP activity, the network neurons are modeled as integrate-and-fire neurons with synaptic currents mediated by AMPA, NMDA and GABA_A_ receptors with biophysically-realistic conductances and time constants (Table S2).

During the simulation, each neuron individually receives stochastic excitatory Poisson inputs from several external sources. The noise fluctuations around the mean external input applied to each neural population thus depend on the amount of neurons in the respective pool (“finite size” effect) and would be zero for an infinite number of neurons. For the two selective populations (consisting of 160 neurons in the present network) the standard deviation is 17 Hz given a total external input of about 2.4 kHz (see below “Input fluctuation analysis”). These 2.4 kHz, equal to 800 afferent neurons firing at 3 Hz, simulate the spontaneous activity in the cerebral cortex outside the local network. On top of this background activity, an external target and motion input are applied to the selective neural populations only (Fig. 1C). They correspond to the sensory stimuli during the RDM experiment: the visually shown target signal and the random-dot motion respectively. In Resulaj's experiment [9] the two possible targets were visible throughout the trial. During neurophysiological single cell recordings combined with the RDM task, one target is always placed in the response field of the recorded LIP neuron. Thus, the selective populations are supposed to respond not only to the motion evidence in favor of one of the two target directions, but also to the targets themselves. The time course of the target input aims to replicate the evolution of LIP firing rates after target presentation. In previous neurophysiological studies [6], [8], [15], LIP firing rates were found to rise steeply with the appearance of the possible targets, followed by a “dip” in activity at the onset of the motion stimulus. Correspondingly, in the simulations the target signal (Fig. 1C, red) is composed of initially high inputs and a subsequent decline of inputs, emulating an attentional shift or upstream inhibition of the target signal at motion-stimulus onset [13], [16].

As LIP neurons are mostly associated with saccadic motor responses, while participants in the experiments of Resulaj et al. [9] performed arm movements, it is worth emphasizing that the neural activity described above and in the following is not confined solely to LIP neurons. Other areas in the posterior parietal cortex (PPC), especially the parietal reach region (PRR), involved in the preparation of arm movements, share those neural characteristics. In particular, neurons in PRR show sustained activity during delayed reach to target tasks and also exhibit huge responses to the appearance of a visual reach target in their response field, very similar in size and time course to LIP neurons for saccades in the same paradigm [17]–[19]. Besides, Cui and Andersen [18] reported that, although generally LIP seems to respond more to eye and PRR more to arm movements if monkeys are free to choose the motor response, a substantial number of LIP neurons responded preferably to arm movements for instructed motor responses. In sum, the assumptions and predictions on neural activity presented in this study apply generally to both LIP and PRR. Note however, that the presented network is generally capable of decision-making and changes of mind even in the absence of a target signal (Fig. S1).

The motion input represents activity of middle temporal (MT) area neurons projecting to PPC. MT neurons fire dependent on the amount of coherent motion towards their preferred direction [20]. Accordingly, the different motion coherence levels are translated into a bias of the motion input to one of the selective populations: for 0% coherence in the random dot motion, both selective pools receive the same amount of motion input (70 Hz, Fig. 1C blue), while for 100% coherence only one pool would receive the maximum motion input (140 Hz). In the following we refer to both the target and motion input as “selective inputs”.

With the start of the motion input the system dynamically evolves towards the decision state, where one of the two selective pools fires at a high rate, the other at a low rate. During this transition, a (first) decision is made when one of the firing rate transients crosses the decision threshold (44 Hz) with the additional condition that the difference between populations is at least 10 Hz. A trial was considered a change of mind, if the firing rate of the initially losing selective pool exceeded the (same) decision threshold after the first pool crossed, and their rates differed again by 10 Hz or more. Our main motivation to use a difference criterion in addition to the fixed threshold was to avoid very occasional joint threshold crossings to count as decisions (see example in Fig. S2E). As fluctuations in the firing rate of the selective populations are rather anticorrelated because of the global feedback inhibition and typically larger than 10 Hz, given the amount of noise present in the network, that constraint has only little effect on the simulation results. In Fig. S2 we show the robustness of our simulation results to variations in the decision criteria.

The motion stimulus in the experiments was turned off when the handle left the starting position. At that point, new evidence that was not taken into account for the first decision could already have arrived in LIP during motor preparation and initiation (∼180 ms [17], [18]). In addition, the last evidence shown to the subject would reach LIP only after a sensory latency of about 200 ms [3], [6]. Taken together, after the first decision, new, yet unprocessed evidence on the motion direction, was possibly available to LIP for a time equivalent to the non-decision time t_ND_ = 380 ms of a trial, i.e. for the duration of motor initiation, plus the latency for the evidence to arrive in LIP. The assumed t_ND_ value of 380 ms for the non-decision time is in agreement with the fit of a simple accumulation-to-bound model to the experimental data of the three participants [9]. Resulaj et al. [9] indeed found, that random fluctuations in the motion stimulus during this time period correlated with changes of mind, indicating that the new evidence caused the subjects to change. In the model, a change of mind without motion input is very unlikely (see below: Verification of mean-field prediction). For computational and analytical reasons (as we were interested in the further progression of the transients to the attractor states), the motion input in the model lasted until the end of the trial simulation (3,500 ms). Therefore, we imposed a timeout of t_ND_ for changing after the first threshold crossing, which implements the experimental time limit for new evidence, caused by switching off the motion stimulus at movement initiation. Note that the simulations are still perfectly congruent with the experiment up to the first threshold crossing plus t_ND_, and also thereafter, as neither in the model nor in the experiment further changes (or threshold crossings) are expected.

### Comparison to behavioral data

**Figure 2:**
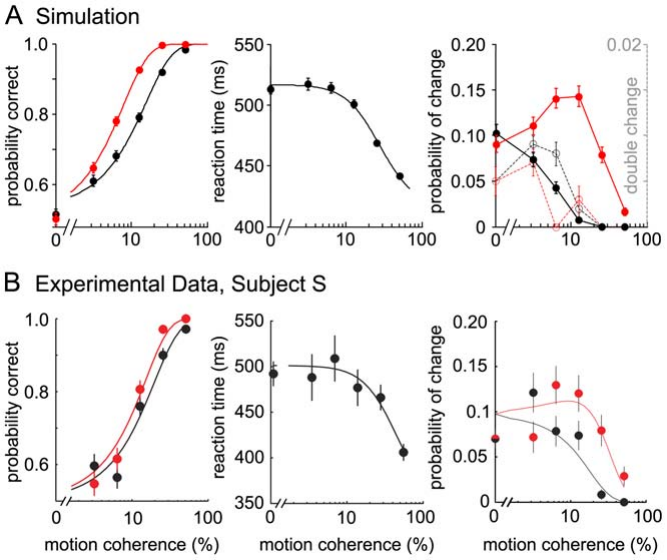
Simulated psychometric functions, reaction times and rates of changes compared to experimental data. (A) Simulation data. The firing rate threshold to determine the first decision (and also a subsequent change) was set to 44 Hz in the simulations. The reaction times include a non-decision time (t_ND_) of 380 ms; t_ND_ also set the time limit for changes of mind. A trial was considered a change of mind, if the firing rate of the initially losing selective pool crossed the decision threshold within t_ND_ after the first crossing of the other pool, and their rates differed by more than 10 Hz. The probabilities of correct responses were fitted to a logistic function, the reaction time to a hyperbolic tangent function. The model parameters were adjusted by hand to fairly fit the average performance of the three subjects that participated in the experiments by Resulaj et al. [9]. For comparison, the experimental performance of one of the subjects (Subject S) is shown in (B) with permission from Resulaj et al. [9]. (Left panel) As in the experimental data, the performance improves through the changes. The first decision (black trace, corresponding to choice at movement initiation) is less accurate than the final choice (red trace, corresponding to the finally chosen target). (Middle panel) The model fits the experimental reaction times well. (Right panel) In the simulations and the experiments, changes to the incorrect choice (black, solid line) decayed monotonically with increasing motion coherence, while changes to the correct choice (red, solid line) peaked at intermediate motion strength and were generally more frequent. Double changes in the simulations are shown on a ten times smaller timescale (right) (open circles, dashed lines). Black (red): proportion of erroneous (correcting) changes that switched a second time. Error bars denote SEM.

**Figure 3:**
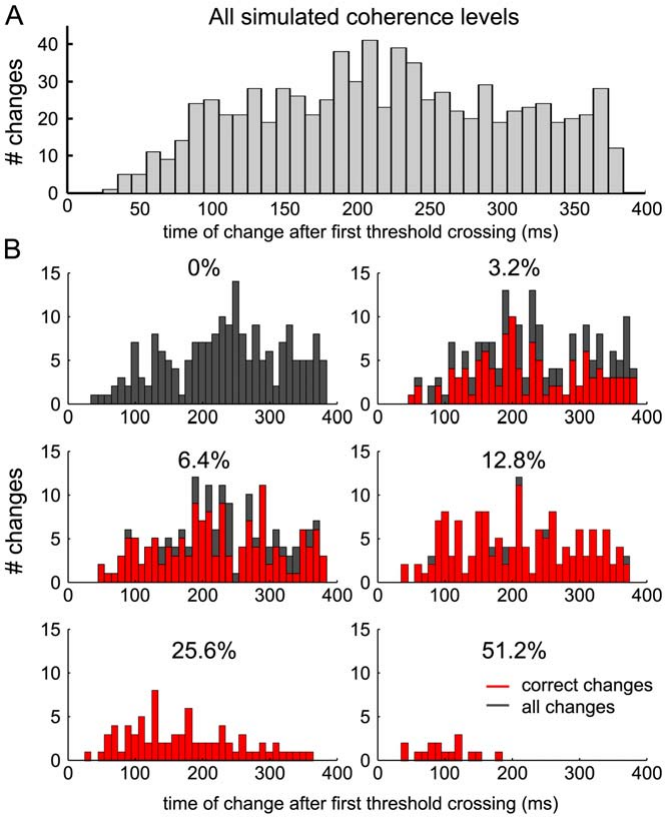
Distribution of change times. (A) Histogram of the time difference between the first and second threshold crossing (change of mind) for all change trials. The change times are broadly distributed from about 50 ms after the first decision to the timeout t_ND_ for changing. (B) Same as (A) separated into coherence levels. All changes are shown in dark grey. The correcting changes are overlaid in red, except for 0% coherence, where changes are neither correcting nor erroneous.

Fig. 2A shows the simulated behavioral data. In the experiments the reaction time was set by the initiation of the hand movement. Accordingly, the simulated reaction time is composed of the time of first threshold crossing, plus the non-decision time t_ND_ = 380 ms. The reaction times and percentages of correct choices fit the experimental results well (Fig. 2, left and middle panel, for further comparison see [9]). Moreover, the model also replicated the frequency of changes observed experimentally (Fig. 2, right panel). Taking the changes of mind into account improves the performance (Fig. 2A, left panel, red line), as changes from wrong to correct choice are more frequent for all coherence levels, but especially for intermediate difficulty. Changes to the wrong alternative, however, are most frequent for low motion strengths and do not occur for high motion coherence. In comparison to the experimental findings, the model predicts slightly more changes to correct and less to the wrong choice, which also explains the larger difference of performance with and without changes (see discussion). Resulaj et al. [9] further noted that a seemingly optimal strategy to opt for or against a change would be to always wait until the end of t_ND_ after the first decision and, thus, to consider all possibly available evidence. This, however, was not consistent with their experimental observations. Along that line, we analyzed the time distribution of changes of mind in the attractor model (Fig. 3). In the simulations, the changes are broadly distributed across t_ND_, with the exception that hardly any changes occur during the first 50 ms after the first decision. The distribution peak depends on the motion coherence level, with earlier changes for higher coherences (Fig. 3B). Interestingly, the time difference between threshold crossings for erroneous changes is not considerably shorter than for correcting changes, although there is more evidence in favor of changing in the case of an initially wrong choice. Erroneous changes just become overall less frequent with increasing coherence.

Moreover, in the simulation in at most 1.6% of the trials two changes occurred during t_ND_ (Fig. 2 right panel, dashed line). The second change was then neglected. Notably, these double-changes were indeed occasionally found in the experiments (M.N. Shadlen, personal communication). In summary, although we did not aim for a perfect quantitative fit to the experimental data, the psychometric functions obtained by our model simulations match the experimental observations very well in all relevant aspects.

### Predictions on neural activity

**Figure 4:**
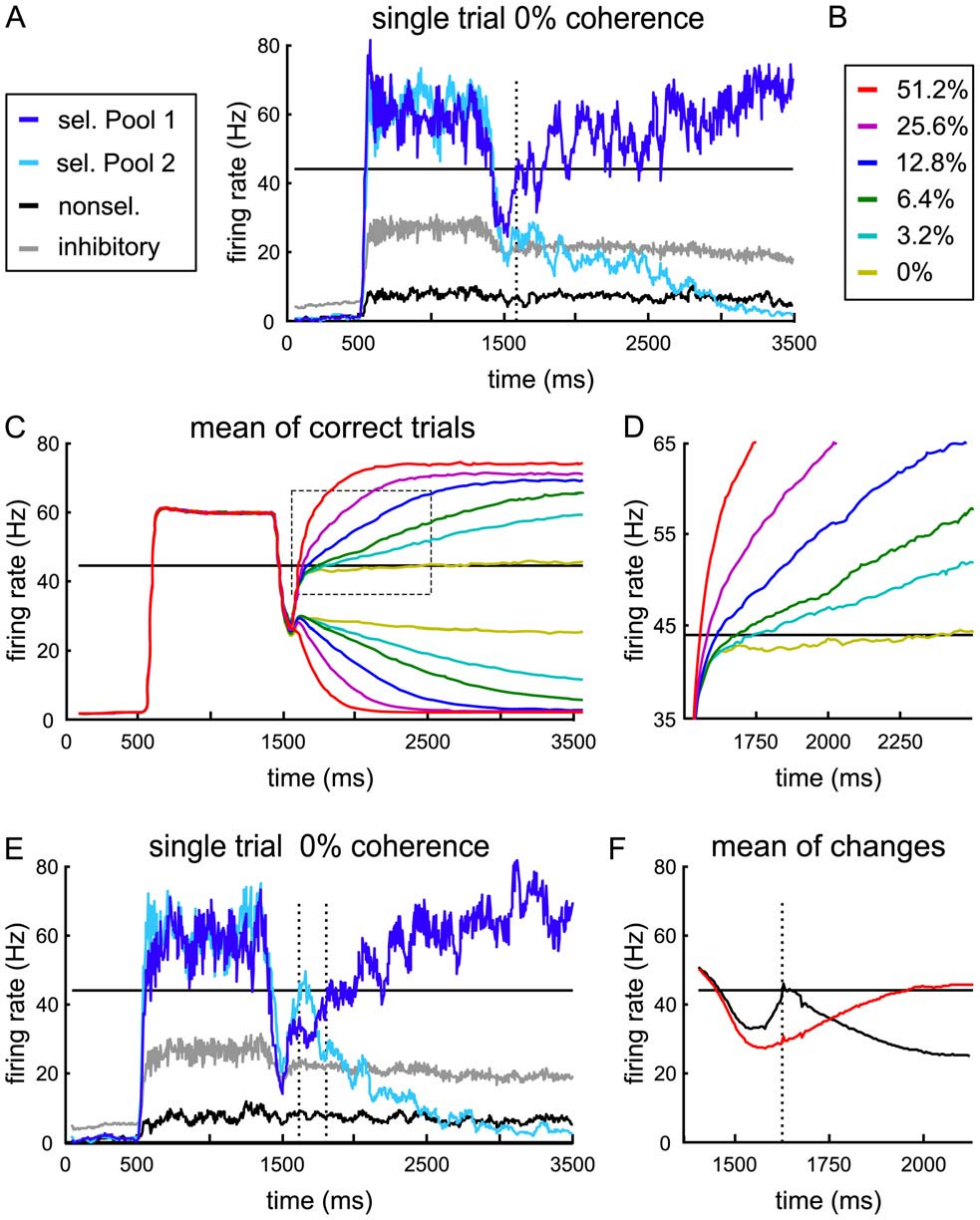
Model prediction of LIP firing rate. (A, E) Simulated temporal evolution of population-averaged firing rates for single trials. The dotted lines mark times of threshold crossings. The black line at 44 Hz indicates the threshold. (A) Example for a regular trial without change. As observed in recent neurophysiological studies of LIP [6], [8], [15], the firing rates of the selective populations show a high increase during target presentation (from 500 to 1,300 ms), followed by a dip after the onset of the motion stimulus. The activities of both selective populations ramp up with the application of the motion input (beginning at 1,500 ms), while the transients compete for the higher attractor state. (C, D) Mean of correct trials from 1,000 network simulations, shown for all motion coherences (Color code according to B). For each motion strength the firing rates were averaged according to the “winners” and “losers” of the first decision. After an initial joint build-up, the slope of the ramping activity is flatter with smaller motion coherence. (D) Blow up of dotted rectangle from (C). (E) In some cases the initially winning population (first threshold crossing) is overtaken by the other transient, which is counted as a “change of mind” trial. (F) Mean of all trials with changes (correct and error trials, all motion coherences) aligned to the first threshold crossing (dotted vertical line). Black: initially winning selective pool, red: finally winning selective pool.

In Fig. 4A and E single trial examples of network simulations are displayed with and without changes of mind. In the trials with identical inputs to both selective pools (0% motion coherence), the decision which population activity will rise or decay is stochastic due to the Poisson inputs and finite-size noise fluctuations. The general temporal structure of the network activity matches single neuron recordings of primate LIP neurons [6], [8], [15] with a high response to the target signals, a subsequent dip of activity and a build-up of the firing rate after the onset of the moving dots, which is steeper with higher motion coherence (Fig. 4C, D average of correct trials at first threshold crossing). Except for the highest motion coherence, this firing rate build-up is biphasic: after an initial steep increase independent of motion strength, the slope of the ramping activity decreases with lower motion coherence. To obtain sufficient changes of mind in the model simulations, the decision threshold was set relatively close to the divergence of the mean build-up activities for different motion coherences, which led to rather small differences in reaction times between the easiest and more difficult trials (see discussion). Nevertheless, the firing rate slopes clearly diverge with motion strength already before the threshold is reached (Fig. 4D).

In Fig. 4F we averaged all simulation trials with changes of mind, aligned to the first threshold crossing, which, if a constant non-decision time is assumed, corresponds to aligning to reaction time in the experiments. Thus, we show that the predicted rise and fall of activity during changes of mind might actually be observed experimentally, even if neural activities obtained in single cell recordings need to be averaged over trials to obtain reliable firing rates. In fact, even for a normally distributed non-decision time with moderate standard deviation, the switch in firing rates should still be discernible in neurophysiological experiments (see Fig. S2H).

### Input fluctuation analysis

**Figure 5:**
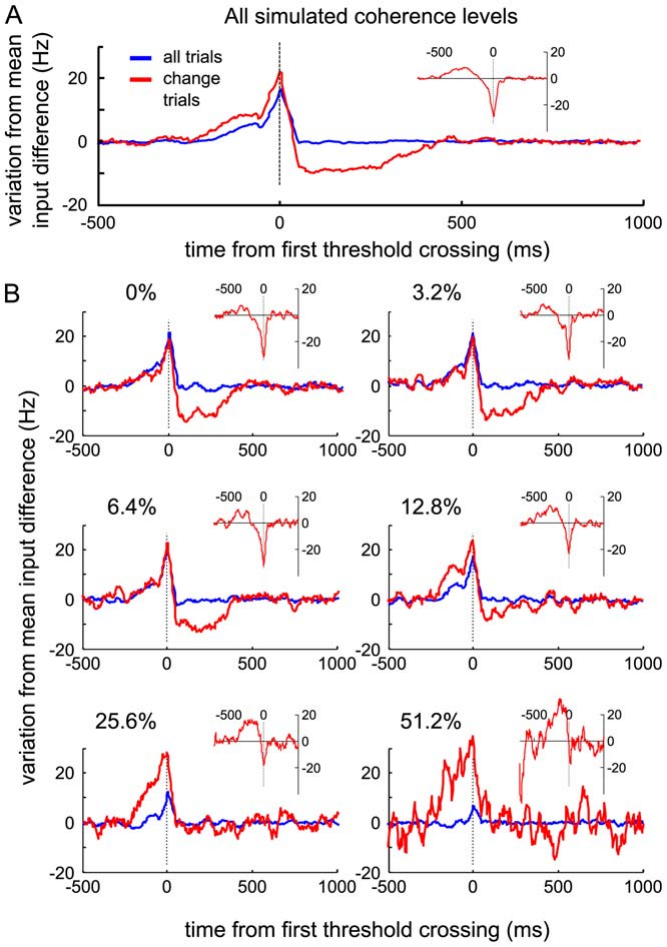
Influence of input noise on changes of mind. The variation from the mean input difference of the selective populations, signed according to which pool first crossed the decision threshold, was averaged, aligned to first threshold crossing, for all trials and all change trials. The insets show the input variation for change trials aligned to the second threshold crossing. (A) Mean across all coherence levels. (B) Separated by motion coherence. Overall and for low coherences, the input fluctuations change sign before a change. For high motion coherence neither correct initial choices, nor changes depend on noise fluctuations (see text).

As most of the dots in the experimental RDM stimulus are moving randomly, the actual momentary level of coherent motion towards the target direction fluctuates around the set mean coherence. A measure of these stimulus fluctuations with respect to the monkeys' choices, the “motion energy”, was found to support the initial decisions as well as the change of mind [9]. More precisely, the fluctuations in the first 150 ms after stimulus onset acted as additional evidence in favor of the first decision (positive motion energy). In change trials the motion energy subsequently became negative, indicating that stimulus fluctuations played a causal role in switching through weakening or even reversing the preceding evidence in favor of the initial choice. In the model simulations, the Poisson noise around the mean input rate corresponds to the experimental stimulus fluctuations. Fig. 5 shows the variation from mean input difference of the selective populations aligned to first threshold crossing and changes of mind (insets). In line with the experimental motion energy, the average input fluctuations across all change trials became negative after the first threshold crossing. Input fluctuations thus act as evidence against the initial choice. Note however, that for high coherence levels the changes do not depend on random fluctuations of the input, since it is mostly initial errors that are reversed by the designated input bias to the correct selective population. Interestingly, the fluctuation strength necessary to reverse a decision is in general not substantially higher than that causing the initial decision.

### Mean-field analysis indicates proximity to bifurcation

**Figure 6:**
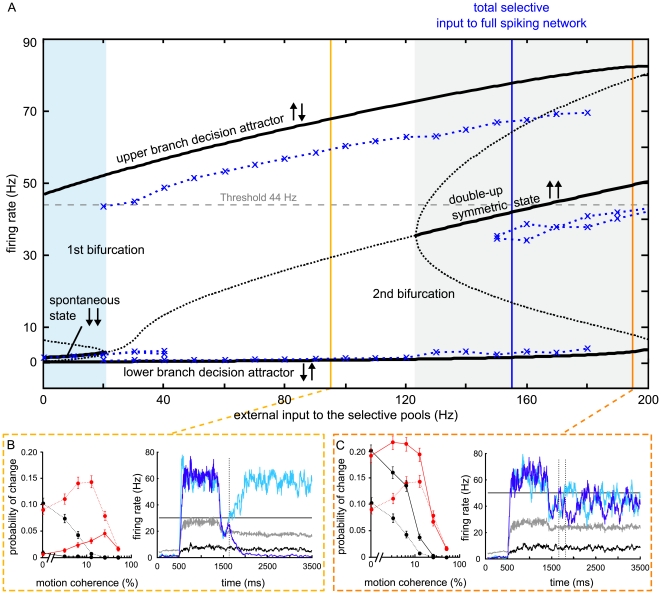
Proximity to bifurcation is important to obtain changes of mind. (A) Mean-field analysis of attractor network. For the parameters used in the spiking model simulation, the stable (solid black line) and unstable (dotted black line) fixed points were calculated with the mean-field approximation over a range of external inputs, applied symmetrically to both selective pools (0% coherence) from 0 to 200 Hz in steps of 1 Hz, in addition to the background input of 2.4 kHz to all neurons. There are three qualitatively different regions to distinguish, separated by bifurcations. In the blue shaded region up to about 20 Hz the spontaneous state (both pools firing at low rates, ↓↓) and the decision state (one pool firing at high, the other at low rates ↓↑) are simultaneously stable. The spontaneous state becomes unstable for higher inputs (white region) until at about 125 Hz a symmetrical state with both pools firing at elevated rates appears (grey shaded area, ↑↑). The blue crosses show the fixed points of the spiking-neuron model for several discrete selective input amplitudes (see methods). The second bifurcation there is shifted by about 25 Hz to higher selective inputs (to the right) for the spiking simulations with respect to the mean-field approximation. The input used in the spiking simulation (blue vertical line, 155 Hz) lies close to the real second bifurcation point. Also, the double-up symmetric state lies below the decision-threshold (44 Hz, horizontal dashed line) while the upper branch of the decision attractor (“winner”) lies above. (B, C) Changes of mind and single trial examples for lower (B) and higher (C) network inputs (yellow and orange lines in (A)). All parameters and the motion input were the same as in the other simulations, only the target input after motion onset was set to 25 Hz for (B) and to 125 Hz for (C). Dashed lines in the left panels give changes of mind from Fig. 2A for comparison. Red: changes to correct, black: changes to wrong choice. With less selective inputs (B), fewer changes of mind are obtained, although the threshold was adapted to fit the reaction times and performance (Fig. S2). With higher selective inputs (C) too many changes are predicted for low motion coherences and the selective transients no longer separate, but stay in the symmetric state. Color of single trial firing rates are the same as in Fig. 3. Error bars denote SEM.

While the model can match the experimentally obtained reaction times and performances for a large range of selective inputs, if the threshold is adapted accordingly (Fig. S3), the feasible range of network inputs is greatly reduced by the additional constraint to match the changes of mind. Using a mean-field approximation of the model [11], we analyzed the dynamical behavior of the network as a function of the selective input amplitude for the parameters that fit the changes of mind. Simulating populations of individual and realistic neurons as described above is necessary to simulate realistic neuronal dynamics, physiological responses and behavior. However, to understand the underlying attractor and dynamical structures prescribing the behavior of population dynamics, we had to use a simpler model that summarized the average activity of these populations. The number of integration variables in the mean-field approximation is reduced to one for each neural population. Thus, it can be solved much more quickly and the parameter space can be scanned (Fig. 6A). Clearly, this obliged us to check the consistence of the mean-field calculations with the simulated activity of the full spiking network. We did this by running both sorts of simulations with the same parameters at key points in their parameter space (see below).

By solving the mean-field equation for a set of initial conditions (here the initial firing rates of each neural population) one obtains the approximated average firing rate of each pool, when the system has settled into a stationary state. These stationary states correspond to the stable states or attractors of the system (Fig. 6A, thick black lines). The unstable fixed points denote the border of the “basins of attraction” of the stable states (Fig. 6A, dotted black lines). The present model has three qualitatively different dynamical regions across the range of symmetric inputs to the selective populations from 0 to 200 Hz, which are separated by fixed-point bifurcations (where a stable fixed point becomes unstable or vice versa). For small inputs the spontaneous state (↓↓), where both selective pools fire at low firing rates, is still stable (Fig. 6A, blue shaded region). At about 20 Hz the system crosses the first bifurcation and the spontaneous state becomes unstable. The network then operates in a region of categorical decision-making, where one selective pool will settle at the upper branch and the other will decay to the lower one. With sufficiently high selective inputs (>125 Hz) a symmetric “double-up” state becomes stable (↑↑), where both selective populations fire with intermediate, elevated rates. Because of the strong recurrent connections within the selective populations, the decision state is stable over the whole range of inputs shown and the spontaneous- and symmetric-state bifurcations are “subcritical pitchfork bifurcations”.

The above conclusions still hold if, instead of symmetric selective inputs as in Fig. 6A, biased inputs are applied, favoring one selective population against the other. In that case the double-up state still exists, but the pool with positive bias will fire at a higher rate than the one with negative bias. The higher the bias, the more will the firing rates of the two selective populations differ in the double-up state. In addition, the basin of attraction of the decision state grows for the favored population at the expense of the other, making wrong choices less likely [16], [21].

The mean-field approximation in general provides an accurate qualitative picture of the attractor landscape. Nevertheless, also quantitative conclusions can be drawn from the analysis. However, there is typically a shift of the predicted fixed-points in comparison to the attractors of the spiking network [11], [22]. To obtain a measure for this discrepancy, we performed network simulations to determine the fixed points of the full spiking model for some discrete selective input amplitudes (see methods), shown as blue crosses in Fig. 6A. At 150 Hz selective inputs the symmetric state was first found to be stable for more than 3,000 ms in 9 out of 100 trials. The real second bifurcation point of the spiking network is thus shifted by about 25 Hz to higher inputs (i.e. to the right) with respect to the mean-field predictions. The input amplitude of the spiking simulation for which changes of mind can be obtained with the attractor model (155 Hz) lies close to this second bifurcation point. Note that in the spiking simulation the dip of firing activity at motion onset marks the start of the transition to the decision state. The initial firing rates of the selective populations (about 25–30 Hz) are therefore located close to the symmetric attractor.

As a consequence of the proximity to the symmetric attractor, the decision process is prolonged [21], [23], making changes of mind more probable. A change of mind is possible until one pool crosses the unstable fixed point (Fig. 6, dotted line between symmetric state and the decision branches) and falls too deep into the basin of attraction of the decision state, where only strong input fluctuations can pull it out again. Taking the shift between the mean-field and spiking-network attractors into account, the decision threshold of 44 Hz coincides approximately with the unstable fixed point and thus with the border between the basins of attraction of the double-up and the decision state. A change of mind can consequently be interpreted as a transient that comes very close to or even surpasses the unstable fixed point, but, because of contrary evidence or fluctuations, does not escape towards the upper decision state and eventually loses the competition.

### Verification of mean-field prediction by spiking simulations

Although the above-presented notion of changes of mind is consistent with the mean-field attractor picture, the accuracy of the approximation is known to be especially weak close to bifurcation points [11], [22]. The mean-field conclusions on the frequency of changes of mind thus have to be validated by simulations with the full spiking network.

Therefore, we performed spiking simulations for all coherence levels for different selective inputs (Fig. 6B, C, yellow and orange lines in Fig. 6A) to further demonstrate the importance of the system's proximity to the symmetric-state bifurcation. All network parameters and the motion input were kept identical to the simulations presented above. The selective inputs were changed by varying the target input after motion onset. The decision thresholds were adjusted so that the model with altered selective inputs fit the experimental reaction times and performances (Fig. S3). For 25 Hz target input (and thus a total selective input of 95 Hz at 0% motion coherence), considerably less changes of mind were obtained, especially for low motion strength, despite the low decision threshold of 30 Hz. By contrast, with a target input of 125 Hz the model predicted too many changes at low motion coherence. More importantly, in most of the low coherence trials with high target input the selective pools did not leave the symmetric state (Fig. 6C, Fig. S3B). Contrary to the concept of using the attractor states to determine the decision outcome, here, even large fluctuations do not necessarily lead to a transition towards the decision attractors. By contrast, close to the bifurcation point, fluctuations will eventually lead to an escape from the symmetric state.

These additional simulations also justify the use of t_ND_ as a timeout for changes: Turning the motion stimulus off with movement initiation would correspond to stopping the motion input in the simulations at t_ND_ after the first decision. The remaining symmetric target input of 85 Hz would be even lower than the selective inputs in the 95 Hz simulations with symmetric inputs (Fig. 5B). Thus, even if changes of mind were possible after t_ND_ they would be very unlikely.

Apart from the input to the selective populations, changing other network parameters will affect the location of the bifurcations. The general shape of the attractor landscape, however, is robust to gradual parameter changes. For example increasing (decreasing) the inhibitory connectivity ω_I_ shifts the whole attractor landscape to the right (left), which has a similar effect as decreasing (increasing) the selective inputs (Fig. 6) and likewise leads to fewer (more) changes (Fig. S4 and S5). This further confirms the crucial role of the symmetric state bifurcation for changes of mind in the attractor network.

### Model predictions on bidirectional random-dot motion

**Figure 7:**
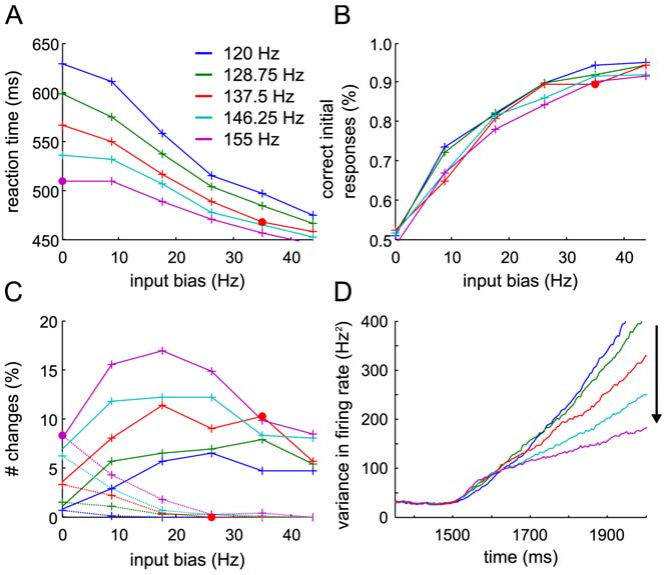
Model predictions for different levels of common selective inputs. The baseline external input, common to both selective populations, as well as the input bias to one of the selective populations were varied in steps of 8.75 Hz. Different colors indicate the amount of common inputs, starting from 120 Hz to 155 Hz (standard input to model the experimental changes of mind). Mean reaction times (A), performance (B) and changes to correct (C, solid lines) and wrong (C, dashed line) alternative are plotted against the input bias between the selective populations. The decision threshold was fixed at the standard decision criteria (44 Hz, 10 Hz difference). 1,000 trials were simulated for each data point. The pink and red dots correspond (approximately) to the standard input parameters used above at 0% and 25.6% (here actually 25%) motion coherence. Increasing the baseline inputs leads to faster reaction times, lower performance and overall more changes. (D) Evolution of the mean firing rate variance across trials for one selective population, starting from shortly before motion input onset (1,500 ms). The firing rate variances increase quite linearly with time. With increasing baseline inputs to both selective populations, the variance across trials becomes lower from ∼150 ms after motion onset.

As shown above, the frequency of changes of mind, as well as the simulated reaction times and performance of the attractor model, depend on the amount of common external input applied to both selective populations (Fig. 6). In Fig. 7 we give a more detailed analysis of simulated behavior with respect to common and biased external inputs, if the decision threshold is fixed at the standard decision criteria (44 Hz, 10 Hz difference). More precisely, we performed additional network simulations starting from various levels of equal external baseline inputs to both selective pools, indicated by different colors in Fig. 7: from 120 Hz in steps of 8.75 Hz to 155 Hz (the standard input close to the second bifurcation, used above to model the experimental changes of mind). On top of that, we varied the bias between the selective populations, again in steps of 8.75 Hz from 0 to 43.75 Hz (abscissa). In this input scheme, the pink and red dots correspond (approximately) to the standard input parameters used above at 0% and 25.6% (here actually 25%) motion coherence. Increasing the baseline inputs leads to faster reaction times and overall more changes. Performance is less affected, but still decreases uniformly regardless of input bias.

**Figure 8:**
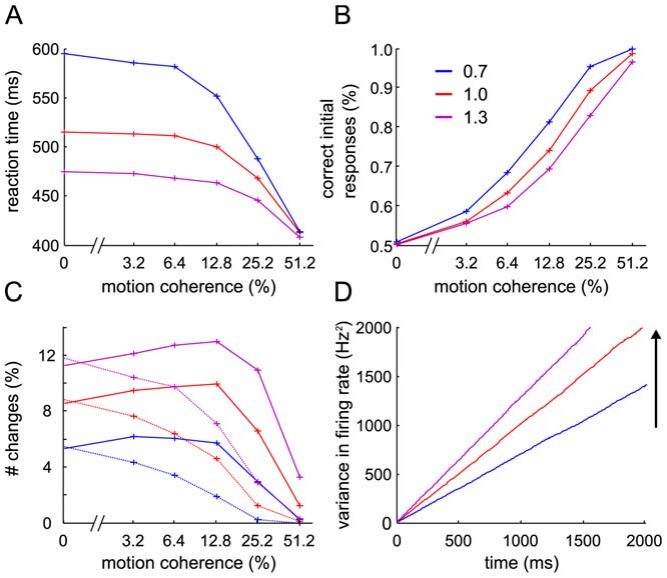
Modifying the variance in the diffusion model. Behavioral predictions of an extended linear accumulator-to-bound model, as used in Resulaj et al. [9] (see methods) for three different levels of input variance (0.7, 1.0, 1.3). Increasing the input variance leads to faster mean reaction times (A), worse performance (B) and more changes of mind (C). (C) Solid lines indicate changes to the correct alternative, dashed lines erroneous changes. 10,000 trials were simulated for each data point. (D) Evolution of the output variance with time. As expected for the diffusion model, the variance rises linearly with time. More input variance leads to more variance across the trials in the output.

An experimental equivalent for higher inputs to both selective populations might be obtained by increasing the overall dot density or with bidirectional random-dot motion, similar to the three-alternative experiment by Niwa and Ditterich [7]. Independent coherent motion in two opposed directions allows comparing differences in the total sensory input while keeping the bias fixed. As an example, in the case of 10% dots moving to the right and 20% to the left, fewer changes, larger reaction times and higher performance would be expected than for 30% of dots to the right and 40% to the left. Such an experiment should generally help to distinguish the nonlinear attractor model from linear diffusion models as used by Resulaj et al. [9], which implement the accumulation of evidence as a single decision variable, encoding only the difference in sensory evidence, but not the absolute value for each direction. Still, changes in the input variance might affect the diffusion model in a similar way as changes in the baseline input affect the attractor network (Fig. 8). Less variance in the input to the diffusion model leads to fewer changes, higher reaction time and better performance. Thus, to unambiguously distinguish the two types of models based on behavioral data, the experimental stimulus fluctuations should be controlled for. Nevertheless, the two scenarios, input variation in the attractor model versus variance changes in the diffusion model, also differ in their predictions on the variance of the output firing rates across trials (compare Fig. 7D with Fig. 8D). While the variance across trials in the diffusion model intuitively increases with increasing input variance, in the attractor model it actually decreases with higher baseline inputs to the selective populations. The reason is again the approximation to the second bifurcation, which impedes the escape to the decision attractors more the higher the inputs, leading to smaller variation in firing rate across trials. Neurophysiological recordings could thus distinguish the two mechanisms based on this higher order measure.

## Discussion

Given the previous success of attractor models to simulate and explain behavioral and neurophysiological data of the RDM task [12], [14], [16] and decision-making in general [2], [24], in this article we made use of a binary attractor model with biophysically-realistic neural dynamics to shed light on brain processes during changes of mind. We showed that, despite their fixed-point stability, attractor models are capable of capturing the essential aspects of changes of mind during the dynamic transitions to the steady states. Moreover, a mean-field analysis revealed that the working point of the network, which fitted the experimentally observed changes of mind, is located close to a bifurcation, where a symmetric elevated state becomes stable. In the following we will discuss this and further model predictions on brain dynamics during changes of mind.

### Distinction against alternative concepts for changes of mind

The presented attractor model offers a simple, yet biologically detailed, explanation for changes of mind with predictions on physiological recordings and the dynamical state of the brain region involved in the decision-making process. As in the bounded-accumulation model of Resulaj et al. [9], a threshold crossing determines the initial choice, which can then be reversed by further processing of the remaining available information. Importantly, the linear accumulator model is not a reduced one-dimensional version of the attractor model. The mechanism behind the changes of mind is quite different. The attractor model is highly nonlinear: once the transient falls into the basin of attraction of the decision state, it is captured by the attractor and a change of mind is no longer possible, except for very strong fluctuations.

#### Comparison with previous studies of the attractor model

The original publication by X.J. Wang [14] discussed decision reversal in the attractor model due to signal reversal, i.e. by explicitly inverting the motion input to the network. Similarly, Wong et al. [16] studied the model behavior if short (100 ms) motion pulses were applied to the selective populations enhancing or weakening the coherent motion. There are two crucial differences between the “changes of mind” observed by Resulaj et al. [9] (which we dealt with in the present study) and the previous approaches on “choice reversal”: first, changes of mind here arise without explicitly inverting the motion evidence, solely by noise fluctuations in the RDM stimulus or, for the simulations, in the external selective input. Second, the inverted inputs in Wang [14] and Wong et al. [16] acted mainly before the decision threshold was crossed a first time and thus affected primarily performance. For a “true” change of mind, i.e. a first decision with a subsequent second threshold crossing, reversing inputs had to surmount the initial motion coherence substantially [14]. In the present study, the input fluctuations inducing changes of mind are of about the same size as the fluctuations preceding the first threshold crossing (Fig. 5). This can be explained by the proximity to the second bifurcation, which delays the ultimate transition to the decision attractors and allows for initial fluctuation in the output firing rate. Changes of mind, without explicitly reversing the input to the selective populations, are therefore not self-evident in the attractor model and occur only rarely, except for the dynamical regime close to the second bifurcation.

#### Comparison with the diffusion model

In order to reduce the free parameters and for physiological considerations, we set the non-decision time t_ND_ as timeout for changing after the first decision and used the same threshold for the first choice and a change of mind. By contrast, Resulaj et al. [9] imposed a second independent threshold and an adaptable timeout for changes to fit their experimental results with an extended diffusion model. Thereby, they could account well for the participants' behavior and the frequency of changes. The predictions on neural activity by the one-dimensional model are, however, quite limited. In turn, we did not attempt a perfect quantitative fit to the data, but provided a neurodynamical explanation for changes of mind, based on the shape of the attractor landscape, which is robust to gradual parameter changes. Still, the simulated behavior fits the experimental data well. The attractor model only predicts slightly less erroneous changes and, hence, a larger difference in performance with and without changes in comparison to the participants' behavior and the diffusion model. This minor discrepancy might be accounted for by modifying the implementation of motion coherence: for simplicity we modeled coherent motion with a balanced input bias that affects both selective populations equally and grows linearly with increasing coherence (see methods, eq. 1). Nonetheless, an unbalanced more positive bias, or a nonlinear increase with coherence (initially less for low coherence and more for higher coherence levels) would be plausible alternatives that could provide a closer fit to the experimental data, without changing any of the predictions or conclusions presented in this study.

Although the validity of the two models cannot be distinguished based on their fits to the behavioral data of Resulaj et al. [9], a slightly modified version of the RDM task with independent coherent motion in two opposed directions [7], which allows comparing differences in the total sensory input while keeping the difficulty fixed, might give more information in that regard. The proposed attractor model predicts that the frequency of changing increases with higher sensory evidence for both alternative directions.

Apart from that, both of the above models assume that the brain continues to process incoming information after the initial decision. This hypothesis still needs to be verified by electrophysiological recordings. Another plausible mechanism is a reset of neural activity after the first threshold crossing. In the attractor model that would cause more changes of mind. This can be understood easily for the 0% motion coherence case: a reset there means starting the decision process from scratch with again equal probability for both choices, while, in order to change decision for continuous processing, the transient first has to escape from the initial attractor. Moreover, resetting neural activity necessarily involves further mechanisms from external brain regions. In this article, however, we aimed to explain the changes of mind as an intrinsic feature of the decision-making process, based on nonlinear evidence accumulation with typical noise fluctuations.

### Two mechanisms for speed emphasis to obtain changes of mind

One requirement for intrinsic changes of mind in the attractor model is a relatively low (first) decision threshold. A low threshold implies fast reaction times and comparatively low performance and thus corresponds to an emphasis on speed against accuracy [10], [25], [26]. Indeed, Resulaj et al. [9] suggest that time pressure induces changes of mind, as fewer changes were observed when participants were instructed to perform more slowly. Moreover, a low threshold in the attractor model leads to the experimental prediction of a bimodal build-up of the mean firing rates (Fig. 4C). After an initial uniform ramping activity that terminates already close to the threshold, the slopes of the average firing rates diverge rapidly for the various motion coherences. As coherence-dependent differences in mean ramping activity only set in near the decision threshold, differences in reaction time with motion strength are rather small. The reaction times of the three participants from Resulaj's experiments are in fact very fast and differ by less than 150 ms between 0% and 51.2% motion strength in comparison to over 400 ms in previous studies with well-trained monkeys [3] or human subjects without explicit instructions on speed or accuracy [10]. More generally, neurophysiological recordings along the lines of our predictions in Fig. 4F could yield further experimental evidence on the existence and value of an absolute decision threshold in LIP.

Apart from the decision boundaries, the speed-accuracy trade-off can, theoretically, be controlled by a second mechanism: Roxin and Ledberg [23] showed that, in a reduction of the attractor model to a one-dimensional nonlinear diffusion equation, higher common inputs to both selective populations lead to a decrease in performance and reaction times (Fig. 7, see further Note 1 in Text S1). Supporting experimental evidence comes from several recent fMRI studies, where an increase in the activity of neural integrators was observed with speed emphasis (reviewed in: [27]). The mean-field analysis and complementary simulations with different selective inputs (Fig. 6) revealed that, in order to explain the frequency of changes found by Resulaj et al. [9], high common inputs to the selective pools are required in addition to a low threshold. Therefore, we suggest that, physiologically, both mechanisms to implement a speed emphasis are essential to explain the experimentally observed changes of mind: high selective inputs and a low decision threshold.

### Physiological relevance of the bifurcation between decision-making and double-up state

Previous analyses of the binary attractor model for decision-making [14], [21], [22] all focused on a region in the vicinity of the first bifurcation, where the spontaneous state becomes unstable. There, performance is high and reaction times are rather long, because of long stimulus-integration times. Recently, also the “double-up” symmetric state gained relevance in connection with target presentation [12], [13], [16], since consistent experimental evidence was found for high firing rates just before stimulus presentation [3], [6], [8], [15]. Assuming high selective inputs with target onset, the double-up state can explain neural activity prior to the decision-making period. Furthermore, in Soltani and Wang [28] cue inputs that arrive while the system is in the symmetric up-state add up to determine the network's starting point for subsequent decision-making, thereby implementing probabilistic inference.

If neural activity in decision-related areas actually evolves according to an attractor landscape, as proposed by this and previous studies (reviewed in: [2]), the dynamical system has to cross a bifurcation in order to switch between the double-up state, effective during target presentation, and the decision-making regime, during random-dot motion. Yet, experimental indications that would suggest any physiological relevance of this second bifurcation for brain dynamics during decision-making have been lacking.

In this study, we found that the attractor model best captures the behavioral data and changes of mind observed in the experiments of Resulaj et al. [9], if the system lies in the proximity of the second bifurcation. We thus proved that all input regimes of the binary attractor model are consistent with particular aspects of the decision-making process and thereby confirmed the suitability of the attractor model to describe neural dynamics. Consequently, we predict that the brain operates over the whole range of inputs that enable decision-making, dependent on the pressure for speed or accuracy, instead of switching between two discrete input levels for decision-making and target representation. This could be tested pharmacologically by gradually blocking inhibition in the decision-related brain areas: decreasing inhibition shifts the working point of the system closer to the bifurcation (Fig. S5). Thus, decreasing reaction times, lower accuracy and more changes would be expected, until the double-up symmetric state becomes stable, where decision making might consequently be impaired completely for low coherence levels.

Taken together, we showed that changes of mind arise naturally in an attractor model of perceptual decision-making by emphasizing reaction speed against accuracy. We suggest that this speed-accuracy trade-off is physiologically implemented by both, threshold adaptation and increasing symmetric inputs. Moreover, we found evidence for the physiological relevance of a so far unregarded bifurcation in the binary attractor model and thereby confirmed the general accordance of attractor networks with neural processes. Finally, we provided predictions on a new experimental paradigm, which might help to distinguish between nonlinear attractor and linear diffusion models.

## Methods

The presented attractor network with biophysically-realistic synaptic dynamics was first introduced to model binary decision-making in [14]. The network kinetics are summarized in Table S1. For details, as well as the mean-field approximation, please refer to the original publications [11], [14] and Suppl. Methods in Text S1. To account for the changes of mind, we adapted the weight parameters and inputs within biologically plausible boundaries (see below). All default simulation parameters are listed in Table S2.

### Neurons and synapses

The network consists of N_E_ = 800 (80%) excitatory pyramidal neurons, N_I_ = 200 (20%) inhibitory interneurons and is all-to-all connected. Single neurons are modeled as leaky integrate-and-fire neurons [29] with conductance-based synaptic responses, characterized by their sub-threshold membrane potential (*V*) dynamics:
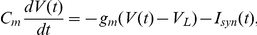
with resting potential *V_L_*, membrane capacitance *C_m_* and membrane leak conductance *g_m_*. A spike is emitted, when the membrane potential reaches the firing threshold *V_th_*. Consequently, *V* is reset to *V_reset_* with an absolute refractory period *τ_ref_*. *I_syn_* denotes the total synaptic current flowing into the cell. It is composed of excitatory recurrent post-synaptic currents (EPSCs), mediated by fast AMPA (I_AMPA.rec_) and slow NMDA glutamate (I_NMDA.rec_) receptors, and inhibitory post-synaptic currents (IPSCs), mediated by GABA_A_ receptors (I_GABA_). External inputs are assumed to be driven only by AMPA receptors (I_AMPA,ext_). In summary:
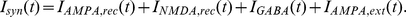
Please see Table S1 for the mathematical description of the receptor kinetics following Brunel and Wang [11]. The parameters for neuronal and synaptic capacities, time constants and conductances are mostly adopted from the original publications [11], [14], except for the recurrent AMPA to NMDA ratio: to better fit the relatively high neural firing rates observed in recent neurophysiological studies [6], [8], [15], we decreased g_NMDA_ by 8% and adapted g_AMPA_ accordingly to preserve the spontaneous spiking rates of about 3 Hz for excitatory neurons and 9 Hz for inhibitory neurons [12].

### Network connectivity

The connections in the network (Fig. 1B) are kept fixed during the simulation and are normalized so that the overall excitatory recurrent synaptic drive remains constant if only baseline input is applied to the network (spontaneous state) [11], by calculating ω_−_ according to 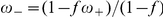, where *f* = 0.2 is the fraction of excitatory neurons in one selective pool, or “coding level”.

### Simulation of sensory inputs

External inputs are modeled as uncorrelated Poisson spike trains. All neurons receive a background input of ν_ext_ = 2.4 kHz, equivalent to 800 excitatory connections from external neurons firing at 3 Hz (Fig. 1B). In the spiking simulation, sensory inputs evoked by the target and motion stimuli (Fig. 1C) are applied (only) to the selective pools. They are present until the end of the simulation (3,500 ms), starting at t_target_ = 400 ms and t_motion_ = 1,300 ms plus an assumed latency of 100 ms and 200 ms, respectively, for the signal to arrive in area LIP [6]. The time course of the target input (Fig. 1C, red) follows the approach of Wong et al. [16]:
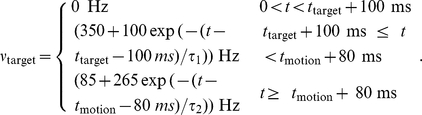
It is in accordance with experimental findings [6], [15] and has since been used in several models of LIP activity during the RDM paradigm [12], [13]. The initial exponential decay τ_1_ = 100 ms can be explained by short term adaptation. Due to the exponential decrease of the target input with τ_2_ = 15 ms, starting with a latency of 80 ms after motion-stimulus onset, the target input is already decaying for 120 ms, before the motion input arrives in LIP with a latency of 200 ms. This causes the dip of firing rate in the simulations. Note that the specific parameters of the target input are irrelevant as long as, first, the initial inputs are high enough to shift the network from the spontaneous to the symmetric state with high firing rates in both selective populations and, second, the target input is reduced sufficiently with motion onset to allow competition (Fig. 5).

The random-dot motion stimulus is simulated as:
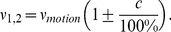
(1)with a time invariant rate of ν_motion_ = 70 Hz for 0% coherence. Coherent motion thus corresponds to a positive bias to one selective pool, balanced by a reduction of the motion input to the other. We simulated six coherence levels: *c* = 0%, 3.2%, 6.4%, 12.8%, 25.6%, and 51.2%.

### Simulations

1,000 trials of 3,500 ms were run for each parameter set and motion coherence. We used a second-order Runge-Kutta routine with a time-step of 0.02 ms to perform the numerical integration of the coupled differential equations that describe the dynamics of all cells and synapses. The population firing rates were calculated by counting all spikes over a 50 ms window and dividing this sum by the number of neurons in the population and the window size. The time window was shifted with a time step of 5 ms. We filtered the external input spikes in the same way to obtain input firing rates for the fluctuation analysis of the external Poisson inputs (Fig. 5). The “variation from mean input difference” was calculated by subtracting the mean input rate across trials from each selective population. The remaining input difference between the selective populations in each trial was then signed with respect to the first pool that crossed the decision threshold.

According to recent experimental findings [3], [6], we assumed a fixed decision threshold independent of motion coherence: a (first) decision was reached when one selective pool crossed a threshold of 44 Hz and surpassed the other by at least 10 Hz. The same conditions applied for a change of mind. To confirm the mean-field approximation (see below), additional simulations were run for different target inputs after motion input onset (25 Hz and 125 Hz instead of 85 Hz), and also for higher and lower inhibitory weights (ω_I_ = 1.425 and ω_I_ = 0.825 instead of 1.125). The respective threshold values were: 30 Hz for 25 Hz target input, 50 Hz for 125 Hz target input, 38 Hz for the simulations with ω_I_ = 1.425 and 50 Hz for ω_I_ = 0.825. All threshold values used were determined within 1 Hz accuracy in order to match the experimental reaction times and percentage of correct choices (A threshold alteration of ±1 Hz roughly corresponds to a ±3% variation in reaction time and about 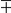
10% in the frequency of changes). For the simulations shown in Fig. 7, the standard threshold parameters were used (44 Hz with 10 Hz difference). The additional condition of a minimal difference of 10 Hz between the firing rates of the two selective populations avoids occasional joint crossings to count as decisions or changes (Fig. S2E). Reaction times were calculated as the time of threshold crossing plus a non-decision time t_ND_ = 380 ms, which consists of a latency of 200 ms for the motion signal to arrive in LIP [3], [6] and 180 ms to account for movement initiation and execution [17], [18]. t_ND_ also set the time limit for the changes of mind. The robustness of the model simulation to variations in the decision criteria and the non-decision time is shown in Fig. S2.

To obtain the stable states of the standard spiking-neuron model in comparison to the mean-field analysis (Fig. 5A, blue crosses), we simulated 100 trials each, without target inputs, but for constant symmetric inputs to the selective populations, ranging from 0 to 200 Hz in steps of 10 Hz for 3,500 ms. The stable fixed points of the decision state were found by averaging the last 500 ms of all trials in which the decision attractor was reached. For (very) low and high inputs, in some (most) of the trials the symmetric spontaneous or double-up state was stable and no decision was formed. The mean firing rate from 1,000 to 2,000 ms of these trials determined the fixed point of the respective symmetric state.

Numerical integration of the mean-field equations was performed using a second-order Runge-Kutta routine with a time-step of 0.1 ms. Stable fixed points were found by terminating integration when the firing rates did not differ by more than 10^−8^ from the mean over the last 40 ms. Unstable fixed points were determined by the boundary of the basins of attraction between two stable states, searched by iterating the initial values between two stable branches to find the change of dynamic flow towards one or the other stable state.

Both, the mean-field analysis and the spiking simulations were implemented in custom-made C++ programs. Custom-made MATLAB programs were used for later analysis, fits of the simulation results and the numerical integration of the diffusion model.

### Diffusion model

The results shown in Fig. 8 were obtained by numerically integrating a diffusion model with an added second threshold and timeout for changing as described in [9]. For the drift and boundary parameters, we used the average fitted value of Subject S from Resulaj et al. [9]: a drift rate 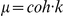, with k = 0.3, a first decision bound B = 13.2, t_ND_ = 324 ms and BΔ = 23.3, without any bias in starting point or drift (μ_0_ = 0, y_0_ = 0). The increments of evidence were obtained from normal distributions with several variance levels. To obtain the predictions on alterations in input variance, we simulated 10,000 trials for each of the six coherence levels, with input variances of 0.7, 1.0 and 1.3, respectively, at time steps of 1 ms.

#### Fits to simulated behavioral data

Psychometric functions (Fig. 2 left panel) were fitted by a logistic function:
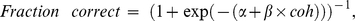
with motion coherence *coh*, *α* and *β* as free parameters. The reaction time curve (Fig. 2 middle panel) was fitted by:
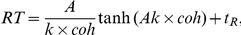
with the free parameters *A*, *k* and *t_R_*.

Error bars denote SEM over all correct trials for simulated reaction times. In the case of probabilities for correct choice and changes of mind the theoretically estimated SEM was calculated according to 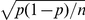 with n = 1,000 trials.

## Supporting Information

Figure S1 Network simulations without target stimulus.
(PDF)

Figure S2 Robustness of simulation results to changes in decision parameters.
(PDF)

Figure S3 Network Reaction times, performance and mean firing rate for different selective inputs.
(PDF)

Figure S4 Spiking simulation with increased inhibition.
(PDF)

Figure S5 Spiking simulation with decreased inhibition.
(PDF)

Table S1 Model summary (according to Guidelines in Nordlie et al. (2009)).
(PDF)

Table S2 Default parameter set used in the integrate-and-fire simulation.
(PDF)

Text S1 Supplementary Notes. Note 1: On the reduction of the attractor model by Roxin and Ledberg (2008). Supplementary Methods. Mean field approximation (following Brunel and Wang (2001)).
(PDF)
