# Supplementary material for: Changes of Mind in an Attractor Network of Decision-Making
Source: PLoS Comput Biol. 2011 Jun 23;7(6):e1002086. doi: 10.1371/journal.pcbi.1002086 (PMC3121686; doi:10.1371/journal.pcbi.1002086)
Supplement: Figure S1 — Network simulations without target stimulus. (PDF) [file pcbi.1002086.s001.pdf]

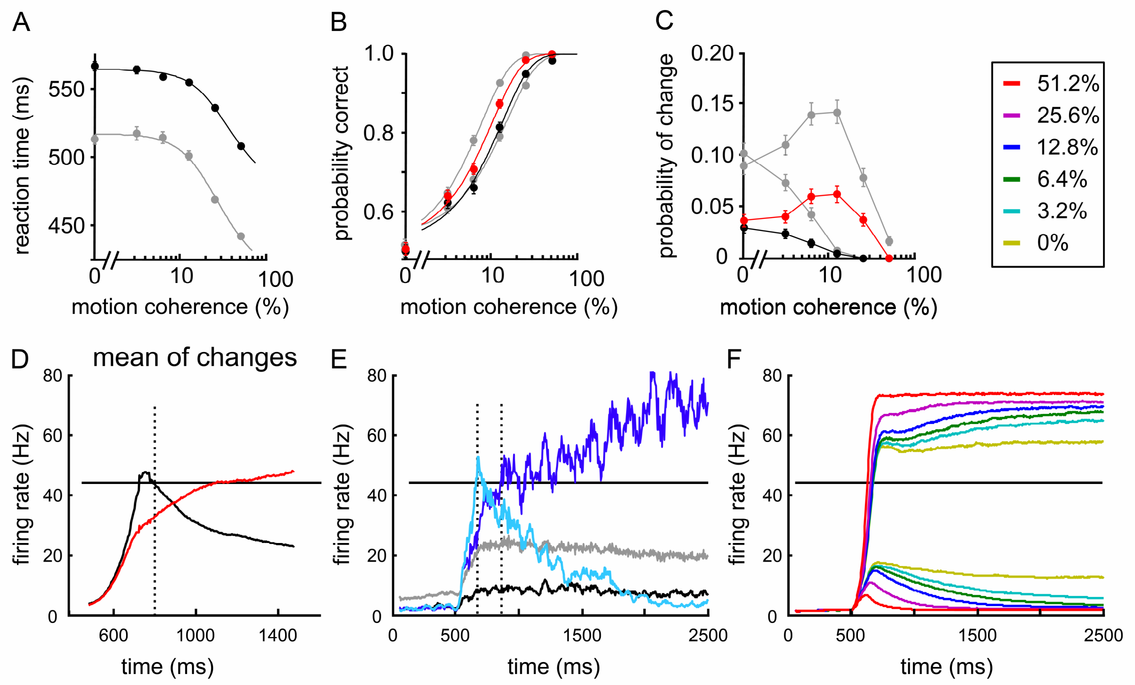

Figure S1: **Network simulations without target stimulus.** To investigate the network's behavior without a time-dependent target signal, we performed additional simulations (1,000 trials for each motion coherence) with the same network parameters as in the main text, but without the initial phase of the target signal. Thus, at 500 ms an input of 85 Hz was applied together with the motion signal (70 Hz ± bias). The network is nevertheless capable of decision-making, although with about 50 ms larger reaction times (A). Results from the main text are shown in gray for comparison. (B) The first-choice performance (black) is unaffected by the missing initial target signal. (C) Changes of mind still occur, albeit fewer, which leads to less performance improvement with changing (B, red). (D) Mean firing rate of changes of mind trials aligned to first threshold crossing. (E) Single 0%-motion coherence trial with change of mind. (F) Mean firing rates of all motion coherences (color legend displayed above). Colors as in Fig. 2 and 3, error bars denote SEM.

The slightly longer reaction times and fewer changes are expected without the high initial target input, as in that case the firing rate transients evolve from the spontaneous state with very low firing rates in both selective populations. Therefore, the transients are more distant to the decision state and double-up state at the start of the motion signal. The match to the experimentally observed reaction times and changes of mind might be improved with threshold adaptation. However, the subjects' behavior is likely to differ as well to some extent, if no targets are presented during the task. Nevertheless, the fact that, even without the initial target signal, changes of mind still occur repeatedly in the model, due to the proximity of the second bifurcation, further strengthens the robustness of our findings.
