# Supplementary material for: Changes of Mind in an Attractor Network of Decision-Making
Source: PLoS Comput Biol. 2011 Jun 23;7(6):e1002086. doi: 10.1371/journal.pcbi.1002086 (PMC3121686; doi:10.1371/journal.pcbi.1002086)
Supplement: Figure S2 — Robustness of simulation results to changes in decision parameters. (PDF) [file pcbi.1002086.s002.pdf]

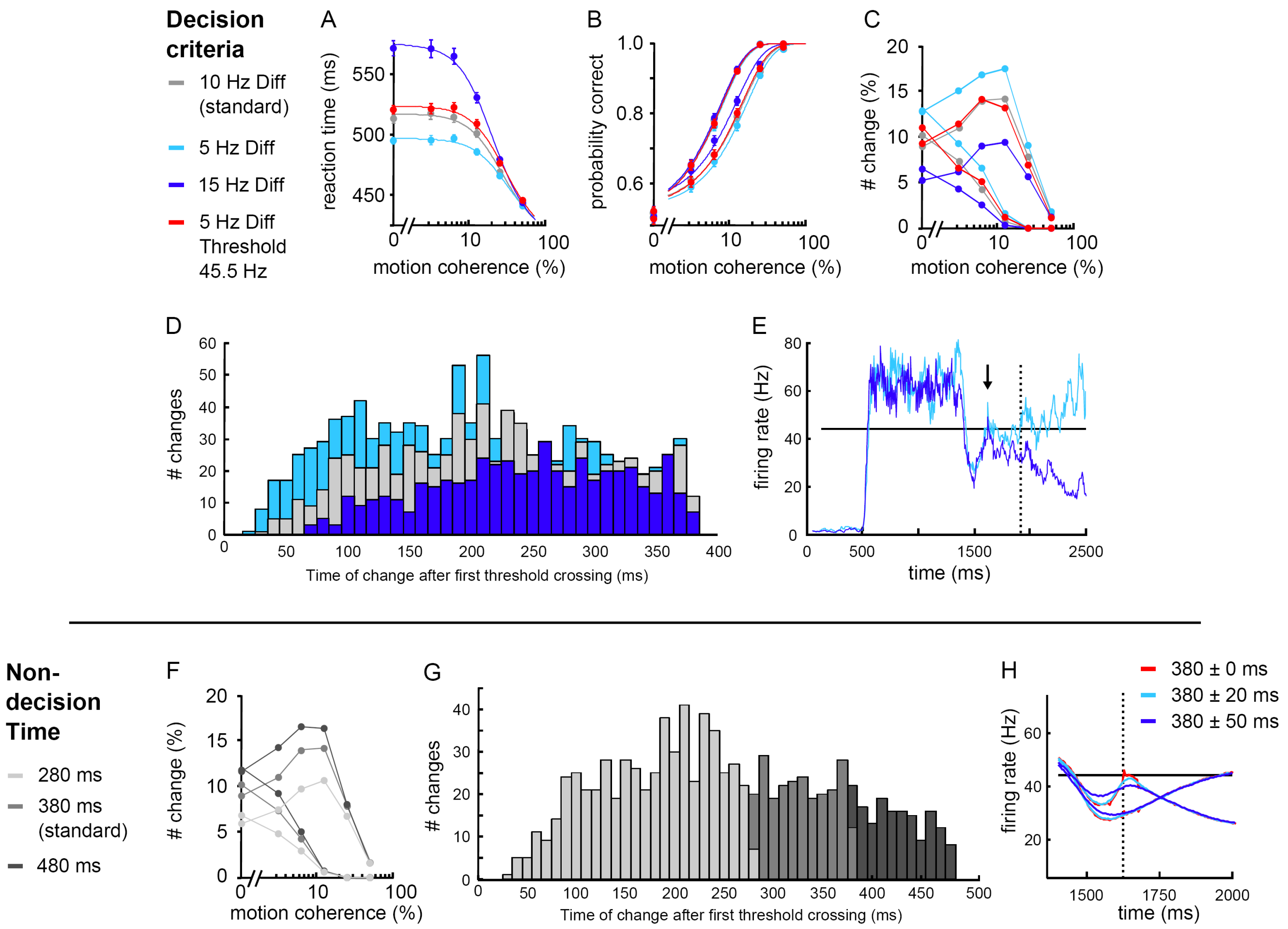

Figure S2: **Robustness of simulation results to changes in decision parameters.** (A-E) Influence of the difference criterion. With respect to the standard decision and change criteria (a 44 Hz threshold and a difference of 10 Hz between the firing rates of the selective populations), a smaller difference criterion (5 Hz) (but the same threshold) leads to faster reaction times and somewhat more changes. A larger difference has the opposite effect. If, in addition to a smaller difference, the threshold is slightly adapted to 45.5 Hz, the results from the standard criteria are fully recovered. (D) The difference criterion affects mostly the amount of early changes, if the threshold is not adapted. (E) Example trial where, during the initial joint increase of activity in the selective populations, both pools briefly cross the decision threshold. Due to the difference criterion this crossing is not taken into account. (F-H) Variability in the

non-decision time. The non-decision time mainly affects changes at low coherences levels. (H) Examples of simulated mean activity for change trials as in Fig. 4F if the non-decision time is drawn randomly for each trial from a normal distribution with mean 380 ms and a standard deviation of 20 ms (light blue) or 50 ms (dark blue). The trials were aligned to first threshold crossing plus the deviation from the mean non-decision time. Even for broadly distributed non-decision times, the switch in firing rates between the two selective populations should still be observable experimentally. Deducing a quantitative value for the decision threshold is however somewhat impeded by a broad distribution of non-decision times.
