# Supplementary material for: Changes of Mind in an Attractor Network of Decision-Making
Source: PLoS Comput Biol. 2011 Jun 23;7(6):e1002086. doi: 10.1371/journal.pcbi.1002086 (PMC3121686; doi:10.1371/journal.pcbi.1002086)
Supplement: Figure S3 — Network Reaction times, performance and mean firing rate for different selective inputs. (PDF) [file pcbi.1002086.s003.pdf]

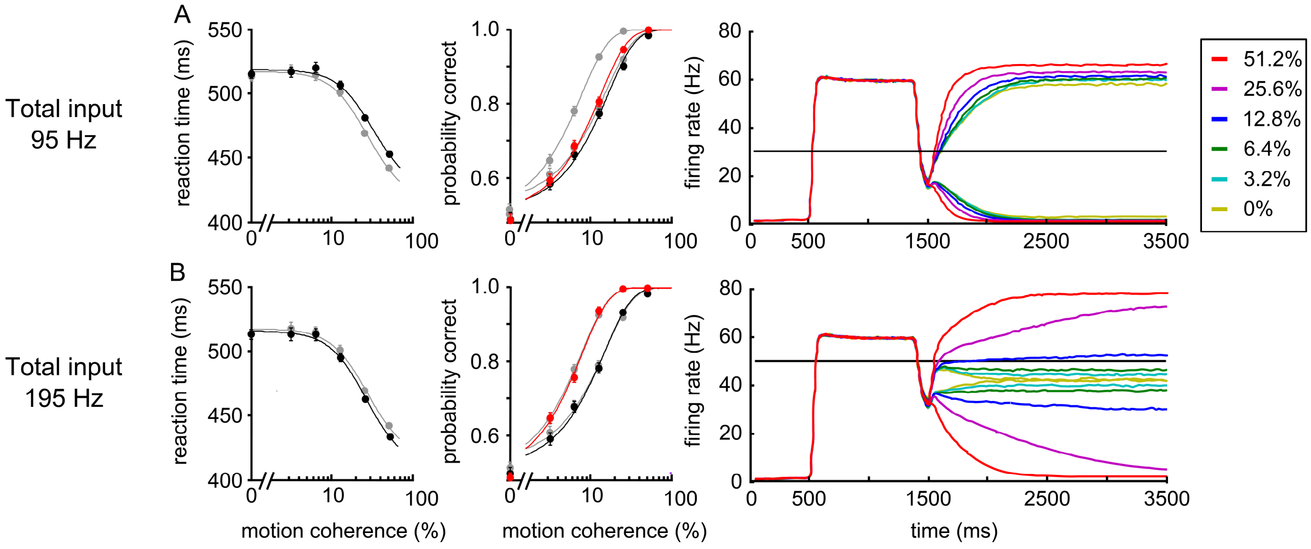

Figure S3: **Reaction times, performance and mean firing rate for different selective inputs.** Colors as in Fig. 2 and 3. (A) With a target input of 25 Hz after motion onset, the threshold has to be lowered to 30 Hz to return approximately the same reaction times and performance as for the standard simulation with 155 Hz of selective inputs and a decision threshold of 44 Hz (grey lines in left and middle panel). As there are only very few changes, the performance with and without changes is very similar. (B) For 125 Hz target input, the threshold has to be increased to 50 Hz to match the values obtained with 85 Hz target input (155 Hz total selective input). Note that the mean firing rates of the correct trials up to 6.4% of motion coherence do not show a build-up to the decision attractor (right panel). A threshold crossing therefore is mainly caused by large fluctuations around the symmetric state and depicts a decision based on evidence integration only for higher motion strength. Error bars denote SEM.
