# Supplementary material for: Changes of Mind in an Attractor Network of Decision-Making
Source: PLoS Comput Biol. 2011 Jun 23;7(6):e1002086. doi: 10.1371/journal.pcbi.1002086 (PMC3121686; doi:10.1371/journal.pcbi.1002086)
Supplement: Figure S4 — Spiking simulation with increased inhibition. (PDF) [file pcbi.1002086.s004.pdf]

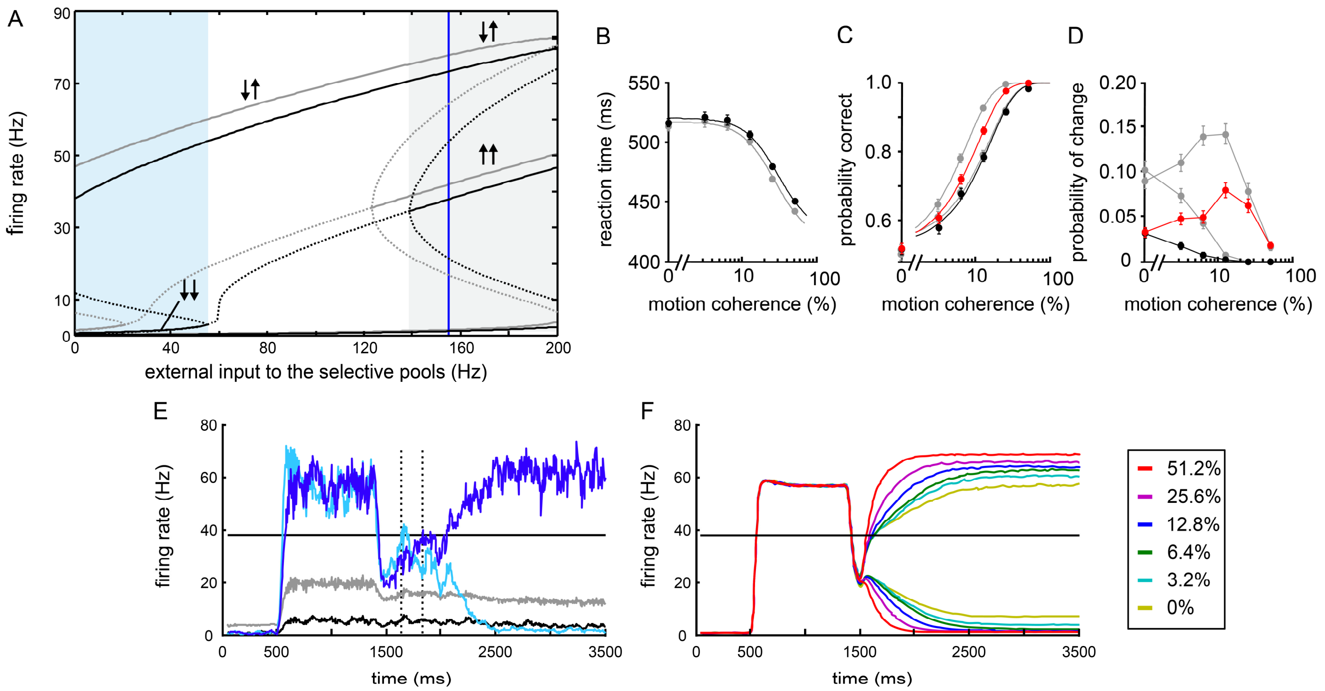

Figure S4: **Spiking simulation with increased inhibition.** (A) Mean-field analysis of network with higher inhibition (ω_I = 1.425 instead of 1.125, all other parameters and inputs as before). Increasing inhibition has similar effects as decreasing the selective input, because the attractor landscape is effectively shifted to the right, towards higher selective inputs. This sets the network input (155 Hz) to the left of the (spiking network) bifurcation point (see Fig. 4A), equivalent to lower inputs with the original inhibitory connection weights. (B-D) The threshold has to be lowered to 38 Hz for comparable reaction times and performance at first choice. Grey lines indicate simulations from the main text with ω_I = 1.125. As for lower inputs (Fig. 4B) increasing the inhibitory connections leads to fewer changes especially at low motion coherence. (E) Single trial example with change of mind at 0% motion coherence. (F) Mean firing rates for correct first choices. Colors as in Fig. 2 and 3, error bars denote SEM.
