# Supplementary material for: Changes of Mind in an Attractor Network of Decision-Making
Source: PLoS Comput Biol. 2011 Jun 23;7(6):e1002086. doi: 10.1371/journal.pcbi.1002086 (PMC3121686; doi:10.1371/journal.pcbi.1002086)
Supplement: Figure S5 — Spiking simulation with decreased inhibition. (PDF) [file pcbi.1002086.s005.pdf]

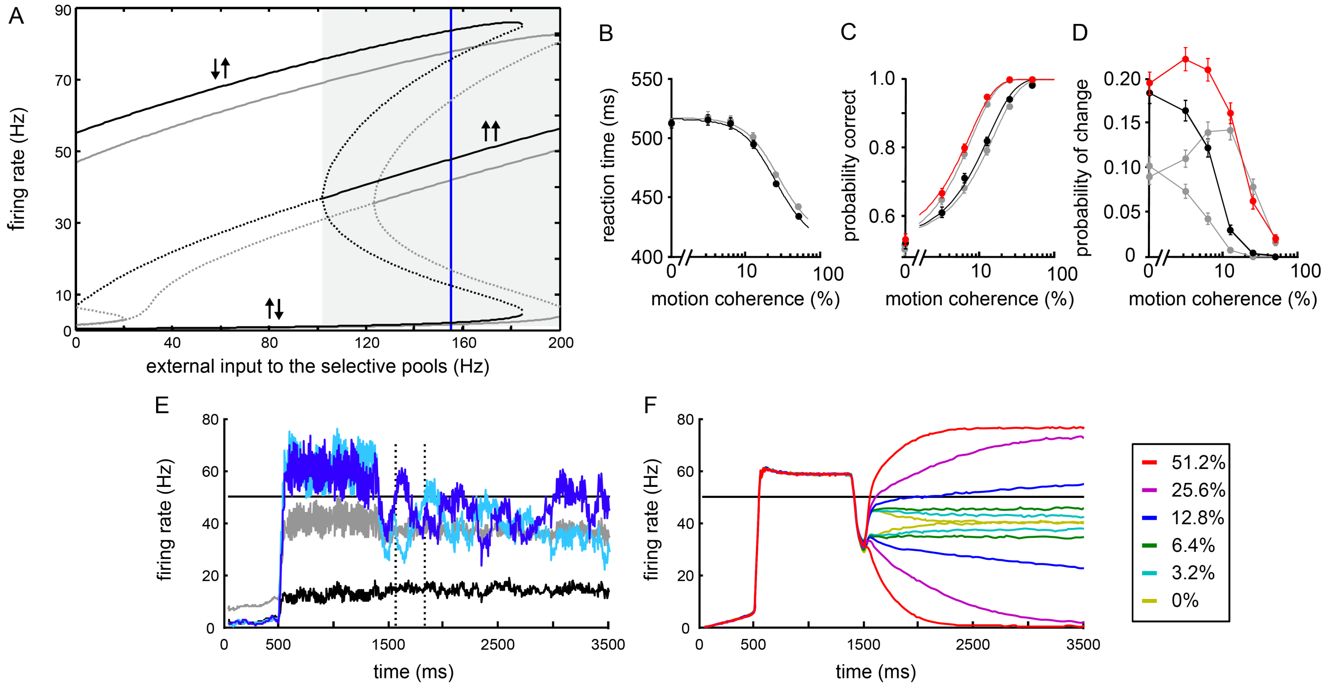

Figure S5: **Spiking simulation with decreased inhibition.** (A) Mean-field analysis of network with higher inhibition (ω_I = 0.825 instead of 1.125, all other parameters and inputs as before). Decreasing inhibition has similar effects as increasing the selective input. The attractor landscape is effectively shifted to the left, towards lower selective inputs. This sets the network input (155 Hz) to the right of the (spiking network) bifurcation point (see Fig. 4A), equivalent to higher inputs with the original inhibitory connection weights. (B-D) The threshold has to be raised to 50 Hz for comparable reaction times and performance at first choice. Grey lines indicate simulations from the main text with ω_I = 1.125. As for higher inputs (Fig. 4B) decreasing the inhibitory connections leads to more changes especially at low motion coherence. (E) Single trial example with change of mind at 0% motion coherence. (F) Mean firing rates for correct first choices. Colors as in Fig. 2 and 3, error bars denote SEM.
