# Supplementary material for: Changes of Mind in an Attractor Network of Decision-Making
Source: PLoS Comput Biol. 2011 Jun 23;7(6):e1002086. doi: 10.1371/journal.pcbi.1002086 (PMC3121686; doi:10.1371/journal.pcbi.1002086)
Supplement: Table S1 — Model summary (according to Guidelines in Nordlie et al. (2009)). (PDF) [file pcbi.1002086.s006.pdf]

Table S 1: Model summary. Parameter values are given in Table S2.

A	Model Summary
**Populations**	Four
**Topology**	One module
**Connectivity**	Full connectivity, no synaptic delay
**Neuron model**	Leaky integrate-and-fire neurons, fixed voltage threshold, fixed absolute refractory periods
**Channel models**	-
**Synapse models**	Conductance-based synapses, AMPA and $\mathrm{GABA_A}$ receptors (instantaneous rise, exponential decay), voltage-dependent NMDA receptors (exponential rise and decay)
**Plasticity**	-
**Input**	Independent fixed-rate poisson spike trains to all neurons
**Measurements**	Spike activity

B		Populations	
Total number of neurons	$N = 1000$	Excitatory neurons	$N_E = 0.8 \cdot N$
		Inhibitory neurons	$N_I = 0.2 \cdot N$
**Name**	**Size**	**Name**	**Size**
Selective pool 1 (right)	$N_{S1} = f \cdot N_E$	Nonselective	$(1-2f) \cdot N_E$
Selective pool 2 (left)	$N_{S2} = f \cdot N_E$	Inhibitory	$0.2 \cdot N$

C				Connectivity			
**Source**		**Target**	**Weight**	**Source**		**Target**	**Weight**
inhibitory	$\mapsto$	all	$\omega_I = 1.125$	nonselective	$\mapsto$	selective	$\omega_- = 0.8725$
excitatory	$\mapsto$	inhibitory	$\omega = 1$	selective i	$\mapsto$	selective j	$\omega_- = 0.8725$
excitatory	$\mapsto$	nonselective	$\omega = 1$	selective i	$\mapsto$	selective i	$\omega_+ = 1.51$

D — Neuron and Synapse Model

Type Leaky integrate-and-fire neurons, conductance-based synapses

Subthreshold dynamics

$$C_\mathrm{m}\dot{V}(t) = -g_\mathrm{m}(V(t) - V_\mathrm{L}) - I_\mathrm{syn}(t)$$

Synaptic currents

$$I_\mathrm{syn}(t) = I_\mathrm{AMPA,rec}(t) + I_\mathrm{NMDA,rec}(t) + I_\mathrm{GABA}(t) + I_\mathrm{AMPA,ext}(t)$$

$$I_\mathrm{AMPA,ext}(t) = g_\mathrm{AMPA,ext}(V(t) - V_\mathrm{E}) \sum_{j=1}^{N_\mathrm{ext}} s_j^\mathrm{AMPA,ext}(t)$$

$$I_\mathrm{AMPA,rec}(t) = g_\mathrm{AMPA,rec}(V(t) - V_\mathrm{E}) \sum_{j=1}^{N_\mathrm{E}} \omega_j s_j^\mathrm{AMPA,rec}(t)$$

$$I_\mathrm{NMDA,rec}(t) = \frac{g_\mathrm{NMDA}(V(t)-V_\mathrm{E})}{1+[\mathrm{Mg}^{2+}]exp(-0.062V(t))/3.57} \times \sum_{j=1}^{N_\mathrm{E}} \omega_j s_j^\mathrm{NMDA}(t)$$

$$I_\mathrm{GABA}(t) = g_\mathrm{GABA}(V(t) - V_\mathrm{I}) \sum_{j=1}^{N_\mathrm{I}} \omega_j s_j^\mathrm{GABA}(t)$$

Fraction of open channels

$$\frac{ds_j^\mathrm{X}(t)}{dt} = -\frac{s_j^\mathrm{X}(t)}{\tau_\mathrm{X,decay}} + \sum_k \delta(t - t_j^k), \quad \mathrm{X} = \mathrm{AMPA}, \mathrm{GABA}$$

$$\frac{ds_j^\mathrm{NMDA}(t)}{dt} = -\frac{s_j^\mathrm{NMDA}(t)}{\tau_\mathrm{NMDA,decay}} + \alpha x_j(t)(1 - s_j^\mathrm{NMDA}(t))$$

$$\frac{dx_j(t)}{dt} = -\frac{x_j(t)}{\tau_\mathrm{NMDA,rise}} + \sum_k \delta(t - t_j^k)$$

Spiking

if $V(t) \geq V_\mathrm{th} \wedge t > t^\star + \tau_\mathrm{ref}$
1. set $t^\star = t$
2. emit spike at time $t^\star$
3. set $V(t) = V_\mathrm{reset}$

E	Input
**Type**	**Description**
Poisson generator	Fixed rate $\nu = \nu_{ext} + \nu_{1,2}$, with ν_{ext} = 2.4 kHz, $\nu_{1,2}$ = sel. inputs, one generator per neuron

F — Measurements

Spike activity: firing-rates were calculated using the spike count in a 50 ms time window shifted by 5 ms steps and dividing by the number of neurons in the population and by the window size.
