# Supplementary material for: Changes of Mind in an Attractor Network of Decision-Making
Source: PLoS Comput Biol. 2011 Jun 23;7(6):e1002086. doi: 10.1371/journal.pcbi.1002086 (PMC3121686; doi:10.1371/journal.pcbi.1002086)
Supplement: Table S2 — Default parameter set used in the integrate-and-fire simulation. (PDF) [file pcbi.1002086.s007.pdf]

Table S 2: Default parameter set used in the integrate-and-fire simulation

Parameter	Value	Parameter	Value
N_{E}	800	N_{I}	200
V_{E}	0 mV	V_{I}	-70 mV
V_{L}	-70 mV	N_{ext}	800
V_{thr}	-50 mV	ν_{ext}	2.4 kHz
V_{reset}	-55 mV	f	0.20
ω_{+}	1.51	ω_{I}	1.125
ω_{-}	0.8725	α	0.5 ms^{-1}
C_{m} (excitatory)	0.5 nF	C_{m} (inhibitory)	0.2 nF
g_{m} (excitatory)	25 nS	g_{m} (inhibitory)	20 nS
τ_{ref} (excitatory)	2 ms	τ_{ref} (inhibitory)	1 ms
$g_{\mathrm{AMPA,ext}}$ (excitatory)	2.08 nS	$g_{\mathrm{AMPA,ext}}$ (inhibitory)	1.62 nS
$g_{\mathrm{AMPA,rec}}$ (excitatory)	0.1872 nS	$g_{\mathrm{AMPA,rec}}$ (inhibitory)	0.1458 nS
g_{GABA} (excitatory)	1.25 nS	g_{GABA} (inhibitory)	0.973 nS
g_{NMDA} (excitatory)	0.30084 nS	g_{NMDA} (inhibitory)	0.23736 nS
$\tau_{\mathrm{NMDA,decay}}$	100 ms	$\tau_{\mathrm{NMDA,rise}}$	2 ms
τ_{AMPA}	2 ms	τ_{GABA}	10 ms
