# Supplementary material for: Changes of Mind in an Attractor Network of Decision-Making
Source: PLoS Comput Biol. 2011 Jun 23;7(6):e1002086. doi: 10.1371/journal.pcbi.1002086 (PMC3121686; doi:10.1371/journal.pcbi.1002086)
Supplement: Text S1 — Supplementary Notes. Note 1: On the reduction of the attractor model by Roxin and Ledberg (2008). Supplementary Methods. Mean field approximation (following Brunel and Wang (2001)). (PDF) [file pcbi.1002086.s008.pdf]

Supplementary Notes

Note 1:

Roxin and Ledberg [1] showed that, in a reduction of the attractor model to a one-dimensional nonlinear diffusion equation, higher common inputs to both selective populations lead to a decrease in performance and reaction times. Strictly speaking, the one-dimensional reduction, and hence also the monotonic dependence of the mean input to speed and accuracy, are analytically only valid close to the first network bifurcation, where the spontaneous symmetric state becomes unstable [1]. In the presented model, the optimal working point of the system in order to account for the experimental data, however, lies close to the other bifurcation, where the symmetric state reappears with elevated firing rates in both selective pools (Fig. 6A). Nevertheless, the mean-field analysis and complementary simulations with different selective inputs (Fig. 6) revealed that, in order to explain the frequency of changes found by [2], high common inputs to the selective pools are required in addition to a low threshold and the monotonic speed-accuracy relation to the selective inputs still holds.

Supplementary Methods

Mean field approximation:

The mean field approximation [3] provides fixed points of the population firing rates, the stationary states of the populations after the period of dynamical transients. In this formulation the potential of a neuron is calculated as:

$$\tau_x \frac{dV(t)}{dt} = -V(t) + \mu_x + \sigma_x \sqrt{\tau_x}\eta(t) \tag{S.1}$$

where $V(t)$ is the membrane potential, x labels the populations, τ_x is the effective membrane time constant, μ_x is the mean value the membrane potential would have in the absence of spiking and fluctuations, σ_x measures the magnitude of the fluctuations and η is a Gaussian process with absolute exponentially decaying correlation function and time constant τ_{AMPA}. The quantities μ_x and σ_x^2 are given by:

$$\mu_x = \frac{(T_{\text{ext}}\nu_{\text{ext}} + T_{\text{AMPA}} n_x^{\text{AMPA}} + \rho_1 n_x^{\text{NMDA}})V_{\text{E}} + \rho_2 n_x^{\text{NMDA}} \langle V \rangle + T_{\text{I}} n_x^{\text{GABA}} V_{\text{I}} + V_{\text{L}}}{S_x} \tag{S.2}$$

$$\sigma_x^2 = \frac{g^2_{\text{AMPA,ext}}(\langle V \rangle - V_{\text{E}})^2 N_{\text{ext}} \nu_{\text{ext}} \tau^2_{\text{AMPA}} \tau_x}{g_m^2 \tau_m^2}. \tag{S.3}$$

where ν_{ext} is the external incoming spiking rate, ν_{I} is the spiking rate of the inhibitory population, $\tau_{\text{m}} = C_{\text{m}}/g_{\text{m}}$ with the values for the excitatory or inhibitory neurons depending of the population considered. The other quantities are given by:

$$S_x = 1 + T_{\text{ext}}\nu_{\text{ext}} + T_{\text{AMPA}} n_x^{\text{AMPA}} + (\rho_1 + \rho_2) n_x^{\text{NMDA}} + T_I n_x^{\text{GABA}} \tag{S.4}$$

$$\tau_x = \frac{C_{\text{m}}}{g_{\text{m}} S_x} \tag{S.5}$$

$$n_x^{\text{AMPA}} = \sum_{j=1}^{p} f_j w_{jx}^{\text{AMPA}} \nu_j \tag{S.6}$$

$$n_x^{\mathrm{NMDA}} = \sum_{j=1}^{p} f_j w_{jx}^{\mathrm{NMDA}} \psi(\nu_j) \tag{S.7}$$

$$n_x^{\mathrm{GABA}} = \sum_{j=1}^{p} f_j w_{jx}^{\mathrm{GABA}} \nu_j \tag{S.8}$$

$$\psi(\nu) = \frac{\nu\tau_{\mathrm{NMDA}}}{1+\nu\tau_{\mathrm{NMDA}}} \left(1 + \frac{1}{1+\nu\tau_{\mathrm{NMDA}}} \sum_{n=1}^{\infty} \frac{(-\alpha\tau_{\mathrm{NMDA,rise}})^n T_n(\nu)}{(n+1)!} \right) \tag{S.9}$$

$$T_n(\nu) = \sum_{k=0}^{n} (-1)^k \binom{n}{k} \frac{\tau_{\mathrm{NMDA,rise}}(1+\nu\tau_{\mathrm{NMDA}})}{\tau_{\mathrm{NMDA,rise}}(1+\nu\tau_{\mathrm{NMDA}}) + k\tau_{\mathrm{NMDA,decay}}} \tag{S.10}$$

$$\tau_{\mathrm{NMDA}} = \alpha\tau_{\mathrm{NMDA,rise}}\tau_{\mathrm{NMDA,decay}} \tag{S.11}$$

$$T_{\mathrm{ext}} = \frac{g_{\mathrm{AMPA,ext}}\tau_{\mathrm{AMPA}}}{g_m} \tag{S.12}$$

$$T_{\mathrm{AMPA}} = \frac{g_{\mathrm{AMPA,rec}} N_{\mathrm{E}} \tau_{\mathrm{AMPA}}}{g_m} \tag{S.13}$$

$$\rho_1 = \frac{g_{\mathrm{NMDA}} N_{\mathrm{E}}}{g_m J} \tag{S.14}$$

$$\rho_2 = \beta \frac{g_{\mathrm{NMDA}} N_{\mathrm{E}} (\langle V_x \rangle - V_{\mathrm{E}})(J-1)}{g_{\mathrm{m}} J^2} \tag{S.15}$$

$$J = 1 + \gamma \exp(-\beta \langle V_x \rangle) \tag{S.16}$$

$$T_{\mathrm{I}} = \frac{g_{\mathrm{GABA}} N_{\mathrm{I}} \tau_{\mathrm{GABA}}}{g_{\mathrm{m}}} \tag{S.17}$$

$$\langle V_x \rangle = \mu_x - (V_{\mathrm{thr}} - V_{\mathrm{reset}})\nu_x \tau_x, \tag{S.18}$$

where p is the number of excitatory populations, f_x is the fraction of neurons in the excitatory population x, $\omega_{j,x}$ the weight of the connections from population x to population j, ν_x is the spiking rate of the excitatory population x, $\gamma = [\mathrm{Mg}^{2+}]/3.57$, $\beta = 0.062$ and the average membrane potential $\langle V_x \rangle$ has a value between -55 mV and -50 mV.

The mean field approximation finally yields a set of n nonlinear equations describing the average firing rates of the different populations in the network as a function of the defined quantities μ_x and σ_x:

$$\nu_x = \phi(\mu_x, \sigma_x), \quad x = 1, ..., n, \tag{S.19}$$

where ϕ is the transduction function of population x, which gives the output rate of a population x in terms of the inputs, which in turn depend on the rates of all the populations.

$$\phi(\mu_x, \sigma_x) = \left(\tau_{rp} + \tau_x \int_{\beta(\mu_x,\sigma_x)}^{\alpha(\mu_x,\sigma_x)} du \sqrt{\pi} \exp(u^2)[1+\mathrm{erf}(u)] \right)^{-1} \tag{S.20}$$

$$\alpha(\mu_x, \sigma_x) = \frac{(V_{\mathrm{thr}} - \mu_x)}{\sigma_x} \left(1 + 0.5 \frac{\tau_{AMPA}}{\tau_x} \right) + 1.03 \sqrt{\frac{\tau_{AMPA}}{\tau_x}} - 0.5 \frac{\tau_{AMPA}}{\tau_x} \tag{S.21}$$

$$\beta(\mu_x, \sigma_x) = \frac{(V_{reset} - \mu_x)}{\sigma_x} \tag{S.22}$$

with erf(u) the error function and τ_{rp} the refractory period which is considered to be 2 ms for excitatory neurons and 1 ms for inhibitory neurons. To solve the equations defined by equation (S.19) for all x, we numerically integrate equation (S.18) and the differential equation below, whose fixed point solutions correspond to solutions to equation (S.19):

$$\tau_x \frac{d\nu_x}{dt} = -\nu_x + \phi(\mu_x, \sigma_x). \tag{S.23}$$

For the numerical integration we used the Heun's method with a step-size of 0.1 ms. To find all the possible fixed points that coexist for a given parameter set, we integrated equation (S.23) with different initial conditions of population firing rates over a range of external inputs ν from 0 to 200 Hz in steps of 1.0 Hz.

References

1. Roxin A, Ledberg A (2008) Neurobiological models of two-choice decision making can be reduced to a one-dimensional nonlinear diffusion equation. PLoS Comput Biol, 4: e1000046.

2. Resulaj A, Kiani R, Wolpert DM, Shadlen MN (2009) Changes of mind in decision-making. Nature, 461: 263-266.

3. Brunel N, Wang XJ (2001) Effects of neuromodulation in a cortical network model of object working memory dominated by recurrent inhibition. J Comput Neurosci, 11: 63–85.
